# Signaling Domain of Sonic Hedgehog as Cannibalistic Calcium-Regulated Zinc-Peptidase

**DOI:** 10.1371/journal.pcbi.1003707

**Published:** 2014-07-17

**Authors:** Rocio Rebollido-Rios, Shyam Bandari, Christoph Wilms, Stanislav Jakuschev, Andrea Vortkamp, Kay Grobe, Daniel Hoffmann

**Affiliations:** 1 Research Group Bioinformatics, Faculty of Biology, Center of Medical Biotechnology, University of Duisburg-Essen, Essen, Germany; 2 Institute of Physiological Chemistry and Pathobiochemistry, Faculty of Medicine, University of Münster, Münster, Germany; 3 Department of Developmental Biology, Faculty of Biology, Center of Medical Biotechnology, University of Duisburg-Essen, Essen, Germany; Max Planck Insititute for Biophysical Chemistry, Germany

## Abstract

Sonic Hedgehog (Shh) is a representative of the evolutionary closely related class of Hedgehog proteins that have essential signaling functions in animal development. The N-terminal domain (ShhN) is also assigned to the group of LAS proteins (LAS = Lysostaphin type enzymes, D-Ala-D-Ala metalloproteases, Sonic Hedgehog), of which all members harbor a structurally well-defined 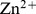 center; however, it is remarkable that ShhN so far is the only LAS member without proven peptidase activity. Another unique feature of ShhN in the LAS group is a double-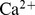 center close to the zinc. We have studied the effect of these calcium ions on ShhN structure, dynamics, and interactions. We find that the presence of calcium has a marked impact on ShhN properties, with the two calcium ions having different effects. The more strongly bound calcium ion significantly stabilizes the overall structure. Surprisingly, the binding of the second calcium ion switches the putative catalytic center from a state similar to LAS enzymes to a state that probably is catalytically inactive. We describe in detail the mechanics of the switch, including the effect on substrate co-ordinating residues and on the putative catalytic water molecule. The properties of the putative substrate binding site suggest that ShhN could degrade other ShhN molecules, e.g. by cleavage at highly conserved glycines in ShhN. To test experimentally the stability of ShhN against autodegradation, we compare two ShhN mutants *in vitro*: (1) a ShhN mutant unable to bind calcium but with putative catalytic center intact, and thus, according to our hypothesis, a constitutively active peptidase, and (2) a mutant carrying additionally mutation E177A, i.e., with the putative catalytically active residue knocked out. The *in vitro* results are consistent with ShhN being a cannibalistic zinc-peptidase. These experiments also reveal that the peptidase activity depends on 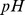.

## Author Summary

Hedgehog proteins are important “morphogens” that steer embryonic development in concentration-dependent ways. Despite many years of intensive research, the mechanism of morphogen action is still under debate. We have studied properties of ShhN, the actual signaling part of Sonic Hedgehog, by a comprehensive set of computational methods and based on experimentally determined molecular structures. Our work suggests surprising new features of ShhN, namely that ShhN is an enzyme that acts as a cannibalistic peptidase, and that this enzymatic function is switched off by the binding of calcium ions to ShhN. Our computational approach reveals the details of this novel switching mechanism. To test the predicted autodegradation of ShhN, we study *in vitro* the stability of specific mutants, and we find that these experiments are in agreement with predictions.

## Introduction

*Hedgehogs* (Hhs) are a conserved family of secreted growth factors essential for development in bilateral animals [1]. Hh proteins realize the so-called *morphogen* principle [2]: they are secreted by specific cells and form extracellular concentration gradients. These are sensed by receiving cells and translated into specific cellular responses in a concentration dependent manner [3]. The basic physical process shaping the concentration gradient is the diffusion of Hh through the extracellular matrix, e.g. in the form of large oligomers [4] or other agglomerates [5]. As altered morphogen concentration may severely affect the cellular responses, the morphogen gradient has to be tightly controlled. Mathematical modeling [6], [7] has pointed to self-enhanced degradation of the morphogen as one possible mechanism to establish the predicted concentration gradients. Molecular feedback loops that lead to self-enhanced removal of Hh, e.g. by receptor–ligand internalization in the target cells, have indeed been found [8]. Another simple molecular implementation of Hh removal would be self-digestion, requiring, of course, that Hh is a peptidase.

In fact, the first crystal structure of the N-terminal domain of Sonic hedgehog (ShhN, the carrier of the morphogen function) revealed that ShhN harbors a 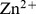 center with a striking similarity to that of the zinc peptidase Thermolysin [9]. Later it was found that the geometry of the ShhN 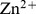 center closely matches a whole class of zinc peptidases [10]: the zinc is tetrahedrally coordinated by 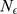 and 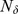 of two histidines, respectively, an aspartate 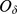, and a water molecule. Moreover, key zinc coordinating residues are attached to a 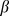-sheet with the same topology in ShhN and all these zinc peptidases. Such a zinc center defines the LAS group of proteins (LAS =  Lysostaphin-type peptidases, D-Ala-D-Ala metallopeptidases, Sonic hedgehog) [10]. For some LAS enzymes even the global fold is similar to ShhN; e.g. the VanX amino-dipeptidase has a root-mean-square deviation of 2.35 Å over 92 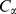 atoms, and its catalytic center maps onto the zinc center of ShhN including the zinc co-ordinating residues and the catalytically active glutamic acid [11]. Yet, although having the same zinc center, ShhN has been the only member of the LAS group without confirmed enzymatic activity.

One confirmed function of the ShhN zinc center is the recognition of cell-surface receptors and their antagonists, though without cleaving these binding partners [12]–[14]. For one of these ligands, Hhip, the binding mode to ShhN was found to be similar to the binding mode of the zinc metalloprotease stromelysin-1 (MMP-3) to its inhibitor TIMP-1 [12], [15] in that the inhibitor completes the tetrahedral co-ordination shell of the zinc ion.

Fuse *et al.* demonstrated that a crucial element of ShhN function is the direct binding to its receptor Ptc [16], but they could not find evidence for peptidase activity in assays with a variety of potential substrates, including some containing D-amino acids (often substrates of LAS enzymes). Given these tests, it is unlikely that ShhN is a broad specificity peptidase such as Thermolysin [17], [18].

A unique feature of ShhN in the LAS group is a second metal ion center with two calcium ions in the vicinity of the zinc center [19]. This double-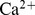 binding site is evolutionary highly conserved and important for the specific recognition of several binding partners of ShhN [12], [13], [19]. In many proteases, 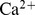 binding activates the enzymatic function. Examples of such 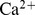 activated proteases with a catalytic 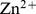 include matrix-metalloproteases [20], Thermolysin [18], or *Helicobacter pylori* metalloprotease [21]. The relevance of 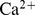 binding in ShhN and other hedgehog proteins is underlined by the strong conservation of this binding site. Consequently, mutations at this site are associated with fatal developmental defects [22]–[24].

In this work we explore possible functions of the metal ion centers in ShhN. One of the hypotheses that are suggested by what we know from other calcium dependent zinc proteases is that ShhN with its LAS peptidase-like 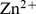 center could be a 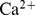 activated LAS peptidase.

To study this hypothesis and to characterize other possible roles of 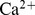 in ShhN, we have investigated by computational means and based on the available ShhN sequences and X-ray structures the effect of metal ions, in particular the effect of 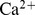 on the structure, dynamics, and electrostatics of ShhN. From these analyses we conclude that 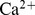 is a regulator of ShhN peptidase, though by an unexpected molecular mechanism. We present *in vitro* experiments testing for the predicted peptidase activity and autodegradation of ShhN by monitoring the stability of specific mutants. These experiments support the hypothesis that ShhN acts as self-degrading peptidase.

## Results/Discussion

### Calcium stabilizes ShhN structure

**Figure 1:**
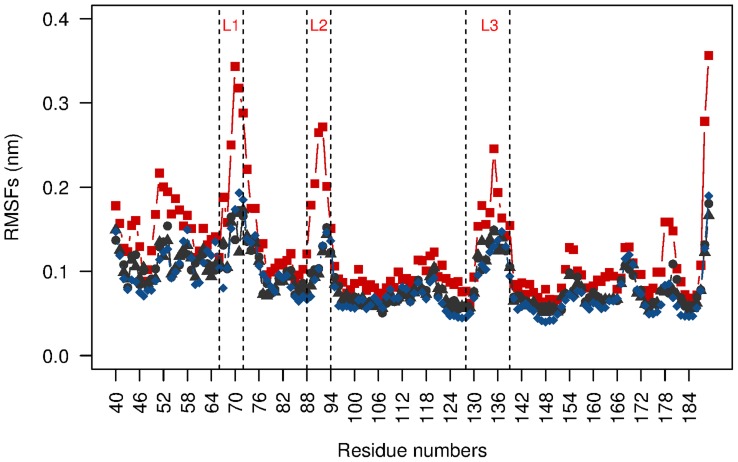
Root mean square fluctuation (RMSF) of protein backbone as function of ShhN residue number. The three calcium binding loops 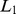, 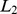, 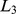 are delimited by vertical dotted lines. Color code as in remainder of paper: state Ca0 without calcium in red (based on PDB entry 1vhh), state Ca1 with one calcium in blue (PDB entry 3n1r), state Ca2 with two calciums in black (based on 3d1m, triangles, and 2wfx, circles).

The first question that we addressed was whether the calcium ions stabilize the structure of ShhN (for the zinc ion see Figure 8 in Text S1). We investigated this question by molecular dynamics simulations of ShhN structures with 0, 1, or 2 calcium ions. Figure 1 shows that the root mean square fluctuation (RMSF, see Methods) of the protein backbone strongly depends on the number of calcium ions. While the states Ca1 (1 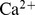) and Ca2 (2 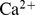) can barely be distinguished, state Ca0 (no 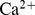) has overall a higher RMSF, especially in the two 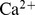 binding loops 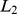 (residues 88–94) and 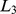 (residues 128–139), and in the neighboring 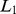 (residues 66–72). The flexibility pattern observed in the molecular dynamics simulations is in good agreement with that derived from crystallographic B-factors (Figure 1 in Text S1) and the experimental observation that 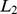, the loop with the highest RMSF values, is disordered in Ca0 [12]. The residue averaged RMSF values are 0.12 nm for Ca0, 0.09 nm for Ca1, 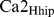, and 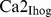 (the latter two based on ShhN complexes with Hhip [12] and Ihog [19]). We applied Wilcoxon tests to all pairs of RMSF distributions, i.e. Ca0 vs. Ca1, Ca0 vs. 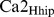, etc., and found that only Ca0 was highly significantly different from Ca1 and Ca2 (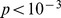, see Table 1 in Text S1), while RMSF distributions of Ca1, 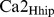, and 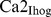 were not clearly different at significance level 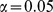. Thus, with respect to flexibility, the binding of the first 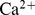 switches ShhN from a more flexible state Ca0 to a more rigid state Ca1, while the binding of the second 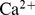 has little impact on flexibility.

**Figure 2:**
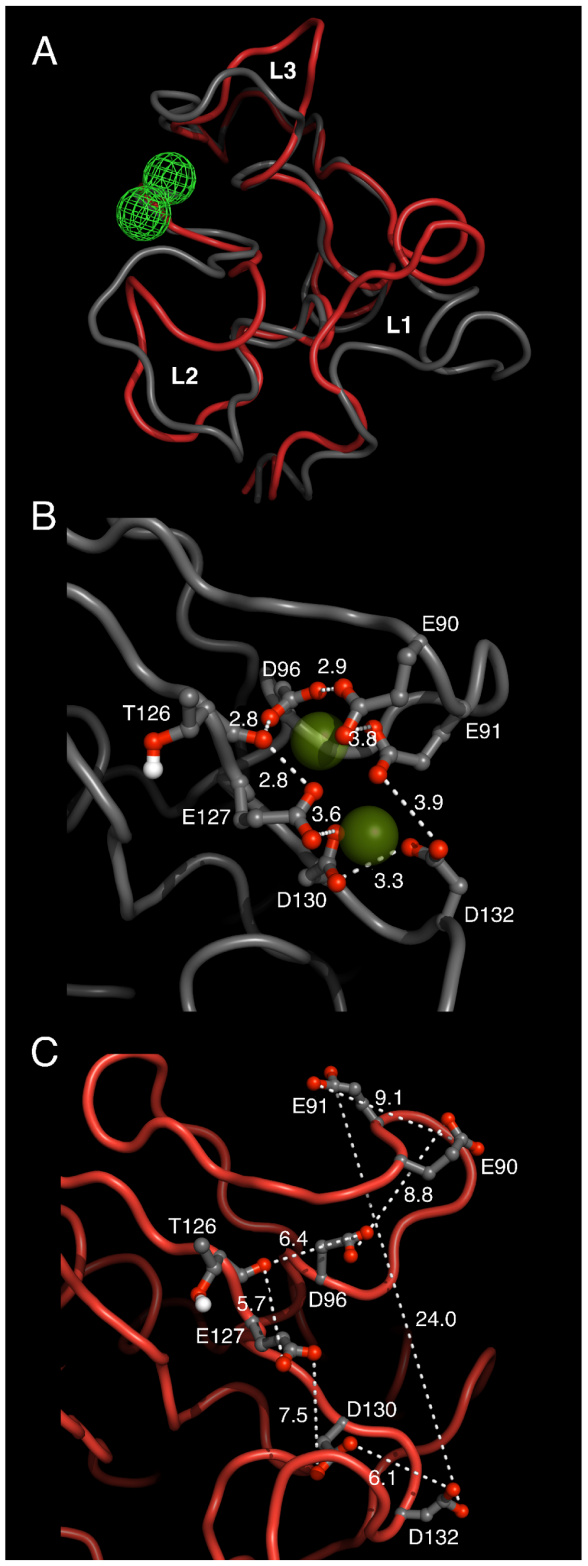
Effect of 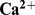 binding on ShhN structure. Structures taken from MD trajectories for Ca2 (grey; based on PDB entry 3d1m with two 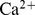) and Ca0 (red; 1vhh). Shown are sampled structures close to the cluster centers of Ca2 and Ca0 ensembles. (A) Overview and comparison of full ShhN structures in Ca2 and Ca0 states. Position of calcium ions marked by green mesh. Calcium binding pocket formed by loops 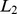, 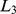 breaks up as 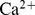 is removed (transition grey to red). Neighboring loop 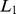 is also affected although it does not co-ordinate 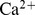. (B) Close-up of 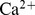 (green spheres) binding site in Ca2 state. Calcium ions are surrounded by a cage of anionic side chains. (C) Same region as in panel B, but now in Ca0 state. Note the large distance differences of anionic groups between B and C.

The transition from Ca2 to Ca0 not only increases the flexibility of ShhN, but induces a different group of structures: the removal of the calcium ions breaks up the binding site and pushes the loops 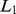, 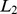, 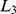, which are located close to the calcium ions, away from each other (Figure 2). This is not surprising as the 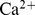 binding sites abound with negatively charged side chains that repel each other.

To further test the stabilizing effect of the calcium ions we simulated the molecular dynamics of states Ca0, Ca1, Ca2 based on X-ray structures of the corresponding states, and measured the root-mean-square deviation (RMSD, see Methods) between initial structures and structures sampled in simulations, expecting to see a higher RMSD for Ca0 and a lower RMSD for the calcium stabilized Ca1 and Ca2. Additionally, we simulated the molecular dynamics of artificial states Ca0, Ca1, Ca2, generated by removing calcium ions from X-ray structures of Ca2 or by introducing calcium ions into the binding pockets in the Ca0 X-ray structure. We found that the RMSDs are consistently higher (usually between 0.15 nm and 0.2 nm) for all Ca0 simulations, and consistently lower (usually 0.15 nm or lower) for Ca2 simulations, irrespective of whether the state was the original one in the X-ray structure, or artificially generated (Figure 2 in Text S1).

### The two calcium ions bind differently

Since ShhN in state Ca0 was found to be more flexible than ShhN in states Ca1 and Ca2, while the latter two had similar flexibility (Figure 1), it seems that the two calcium ions are not equally important for structural stabilization. A closer inspection of the structure shows that, while one calcium is more exposed to the solvent (in Figure 2 close to D132), the other is almost completely engulfed by its ligands (in Figure 2 below E90). The latter calcium ion is the only one present in PDB entry 3n1r that was used for the simulation of the Ca1 state (blue trace in Figure 1). In the following we call this the Ca1 calcium, while the more solvent exposed calcium ion that completes the Ca2 state is called Ca2 calcium.

The observation of structure 3n1r with a single calcium at Ca1 position and a missing Ca2 ion suggests that Ca1 is the ion with the higher affinity. This is consistent with the smaller solvent accessible surface of the Ca1 ion, as there is a negative correlation of calcium affinity and solvent accessible surface [25]. Based on this empirical correlation, we obtain a rough estimate of 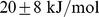 for the difference between the affinities of Ca2 and Ca1 (see Materials and methods).

This ranking of affinities is also in agreement with the 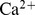 concentrations applied in crystallization and the correspondent Ca1 and Ca2 occupancies: For the crystallization of Ca2 structures 3d1m [19], 3mxw [14], and 2wfx [12], 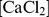 of 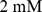, 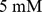, and 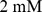, were applied respectively, while the only Ca1 structure 3n1r [26] had a 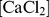 of 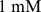. McLellan *et al.* [19] attempted to measure Ca1 affinity and estimated that it should lie above 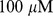. These experiment based values narrow the range of Ca1 affinity to about 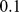 to 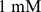 (or 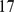 to 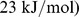. Together with our estimate of the affinity difference between Ca1 and Ca2 of 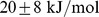 this makes clear that Ca2 is very weakly bound.

Naturally, the question arises whether binding of the Ca2 calcium ion has specific effects on the structure and function of ShhN. A candidate mechanism could be the activation of the Thermolysin-like or LAS zinc peptidase function by binding of the Ca2 calcium ion.

### Ca2 calcium ion changes conformation of putative catalytic center

**Figure 3:**
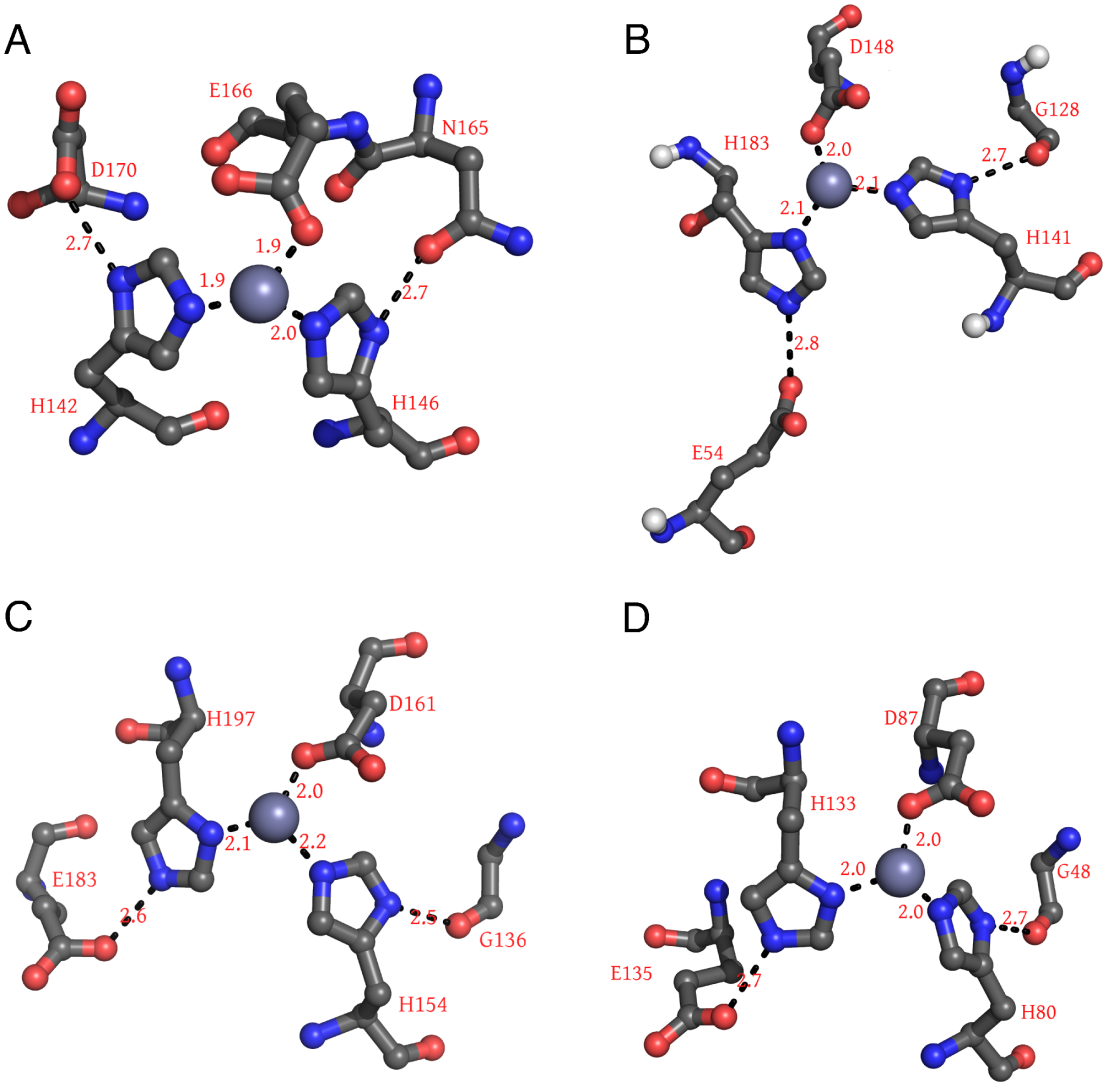
Zinc centers of peptidases and ShhN. The four examples shown are Thermolysin (A), ShhN (B), and LAS peptidases *Streptomyces albus G* D-Ala-D-Ala Carboxypeptidase (PDB: 1lbu) (C), and L-alanoyl-D-glutamate endopeptidase of a bacteriophage (PDB: 2vo9) (D). Numbers on dashed lines are characteristic distances in 0.1 nm.

In the following we therefore focus on the putative catalytic zinc center and investigate the effect of 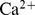 binding on its structure. Figure 3 shows zinc centers of several peptidases known from earlier studies [9], [10] for their high similarity to the zinc environment of ShhN. In all structures, the zinc ion is co-ordinated by nitrogens of two imidazole rings contributed by histidines, and a carboxylate group from a glutamate or aspartate residue. Both imidazole rings are fixated by hydrogen bonds between their N–H groups and neighboring hydrogen bond acceptors. In all four cases, one of these acceptors is a carbonyl oxygen, the other a carboxylate group.

**Figure 4:**
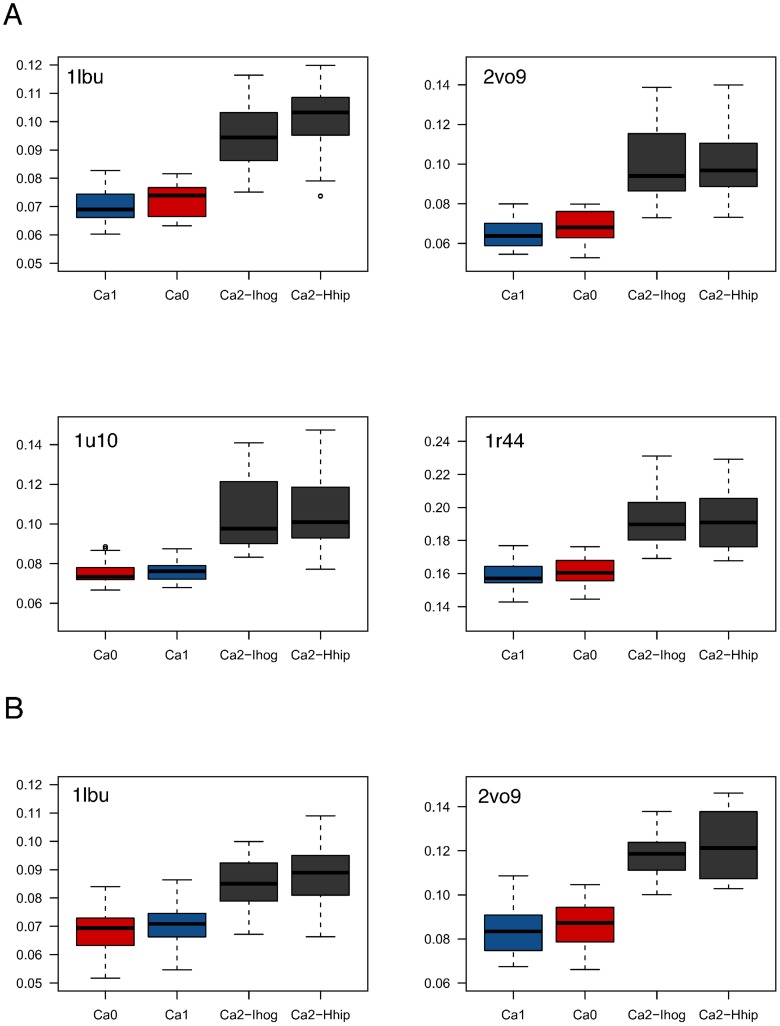
Statistical comparisons of zinc center geometries. (A) RMSDs between zinc centers from LAS enzyme X-ray structures to ShhN in states Ca0 (red), Ca1 (blue), Ca2 (grey) sampled by molecular dynamics. (B) RMSDs between zinc centers from MD simulations of LAS enzymes and MD simulations of ShhN in states Ca0, Ca1, Ca2.

We first hypothesized that addition of the Ca2 calcium makes the zinc center of ShhN more similar to the zinc center of other LAS peptidases. To test this hypothesis, we simulated the molecular dynamics of ShhN in states Ca0, Ca1, and Ca2 and compared the conformations of the zinc environments sampled in this way with the X-ray structures of LAS peptidases with PDB entries 1lbu, 2vo9, 1u10, and 1r44 (Figure 4A). Contrary to our hypothesis, the median of the RMSDs between the sampled zinc center conformations and the LAS peptidase zinc centers were significantly *higher* in the Ca2 state. In other words: the binding of the second calcium makes the ShhN zinc center *less* similar to a LAS enzyme zinc center.

The differences shown in Figure 4A between Ca0 and Ca2, and between Ca1 and Ca2 are significant (significance level of 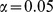, see Table 2 in Text S1). The differences between Ca0 and Ca1 are *not* significant (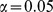). This means that while the Ca1 calcium ion is responsible for stabilizing the overall structure as described in the previous sections, the binding of the Ca2 calcium ion switches the zinc center from a LAS enzyme conformation to a significantly different conformation.

Although this pattern was consistent across the tested LAS enzyme structures, it could theoretically be an artifact related to the comparison of LAS enzyme X-ray structures and ShhN molecular dynamics simulations. To exclude this, we simulated the two LAS enzyme structures (PDB entries 1lbu, 2vo9) that according to their X-ray structures had zinc centers geometrically most similar to the ShhN zinc center. We then compared zinc center structures sampled by molecular dynamics simulations for both the LAS enzymes and the ShhN states (Figure 4B). We found that ShhN zinc centers in Ca0 and Ca1 were significantly more similar to LAS zinc centers than ShhN zinc centers in Ca2 (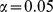; p-values in Tables 6 and 7 in Text S1). Ca0 and Ca1 did not differ significantly in this respect, neither did different versions 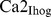, 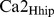. This result agrees with the previous one, again suggesting that binding of Ca2 calcium switches the zinc center of ShhN from a LAS enzyme conformation to a significantly different conformation.

### Switch mechanism

**Figure 5:**
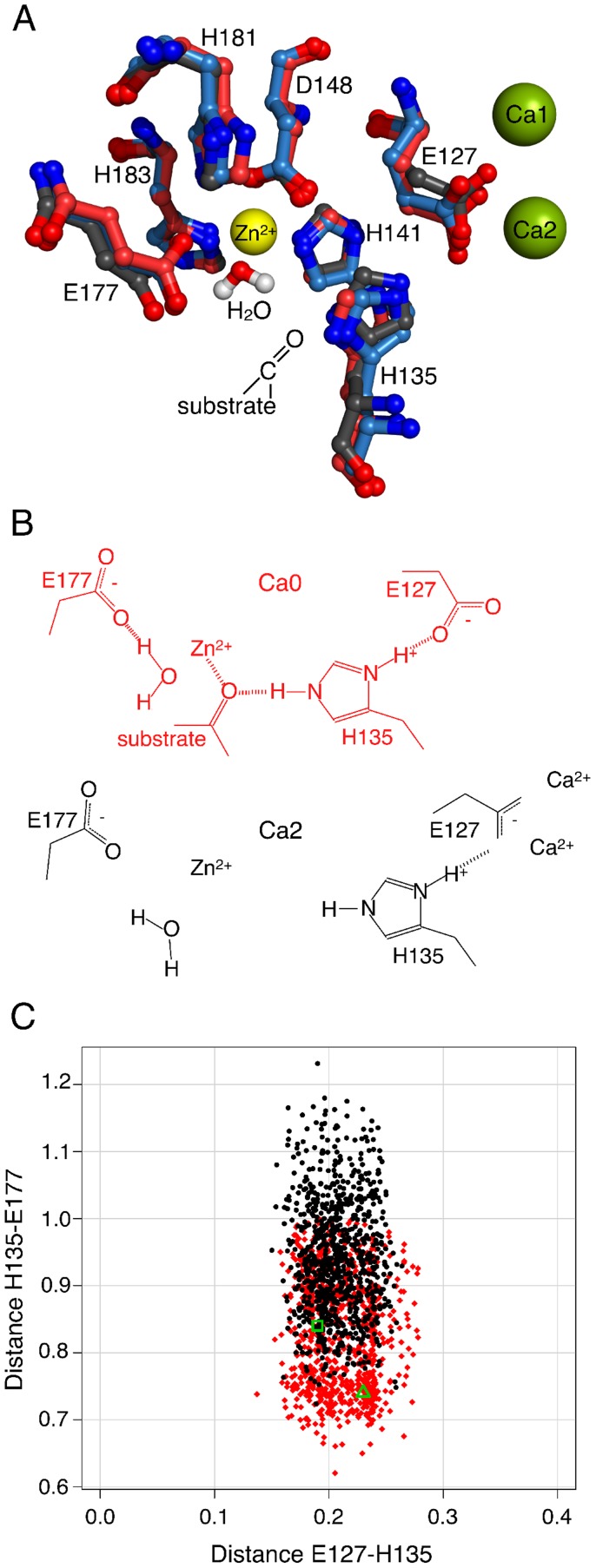
Switch mechanism triggered by Ca2 calcium ion. (A) ShhN zinc center in states Ca0 (X-ray structure 1vhh, red), Ca1 (X-ray structure 3n1r, blue), Ca2 (X-ray structure 3d1m, black). Putative catalytic water from 1vhh is close to the zinc ion. From Ca0 to Ca1 and Ca2, E127 carboxylate is drawn towards 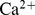 and drags H-bound H135 side chain with it, away from substrate and the active E177. While Ca0 and Ca1 superimpose well, Ca2 is clearly different. (B) Central components of the switch mechanisms in states Ca0 and Ca2. (C) Distances between H-bonded E127 carboxylate-O and H135 imidazole-proton, and between substrate-clamping side chains of H135 and catalytically active E177. Red (Ca0) and black (Ca2) points are sampled by MD simulations. Green triangle (Ca0) and green square (Ca2) are the corresponding values directly taken from X-ray structures 1vhh and 3d1m, respectively.

By which mechanism does the binding of the Ca2 calcium switch the zinc center, which lies 1 nm away? The authors of the first X-ray structure [9] had proposed for ShhN a peptidase reaction mechanism closely related to that of Thermolysin, involving seven residues and the zinc ion. They were unaware of the fact that ShhN binds 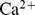 in two well-defined binding pockets. Figure 5A shows the residues of the putative catalytic center in states Ca0, Ca1, Ca2 as obtained by X-ray crystallography, and, for orientation, the positions of the calcium ions. According to the postulated reaction mechanism, E177 abstracts a proton from the catalytic water at the fourth tetrahedral co-ordination site of the zinc ion, followed by a nucleophilic attack of the 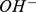 on the substrate carbon.

Amongst the residues in the putative catalytic center, E127 is directly affected by 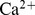 binding as it co-ordinates both the Ca1 and the Ca2 calcium ions. The X-ray structures (Figure 5A) show that, as the Ca2 calcium ion binds, the carboxylate of E127 is markedly drawn towards this calcium ion. Further, there is a hydrogen bond between E127 and H135 observed in the X-ray structures and MD simulations of all three calcium binding states. Hence, as E127 is dragged towards the calcium center, it pulls H135 with it. According to Ref. [9], the second N–H of the H135 imidazole could stabilize the peptidase substrate in a conformation amenable to hydrolysis by forming a hydrogen bond with the carbonyl-O of the substrate. In Ca2, with H135 pulled away, the substrate stabilization in this critical conformation will be affected.

Opposite to H135, at the other side of the substrate, lie the zinc bound water molecule and E177, the two actually catalytically active components according to the enzymatic model in Ref. [9]. As the Ca2 calcium ion is introduced, the gap between H135 and E177 widens by about 1 Å according to the X-ray structures (Figure 5B). This observation is in agreement with the previous one that the binding of the Ca2 calcium ion is accompanied by a significant perturbation of the putative catalytic center, possibly affecting substrate stabilization.

This pattern of conformational change due to the binding of the Ca2 calcium ion is corroborated by the analysis of the MD trajectories of the different states. Figure 5C compares MD simulations of Ca0 and Ca2. It shows that the hydrogen bond between E127 and H135 is conserved between the 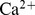 binding states, despite the shift of E127 from Ca0 to Ca2 due to its attraction to the calcium center. Conversely, from Ca0 to Ca2 the distance widens between H135 and E177, thus opening the tight clamp previously gripping putative substrate and catalytic water. Moreover, the variance of this distance increases with the binding of the Ca2 calcium. Numerical details characterizing the distance distributions are reported in Tables 8 and 9 in Text S1.

The pulling of E127 percolates towards the zinc center also along another route. In the description of the zinc center we have mentioned that the zinc co-ordinating histidines are stabilized by hydrogen bonds with carbonyl and carboxylate groups (Figure 3). One of these carbonyl groups comes from the backbone of G128, the sequence neighbor of the 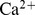 co-ordinating E127. In Ca0 and Ca1, the G128 carbonyl forms a hydrogen bond with the zinc co-ordinating H141. In Ca2, as E127 is pulled towards the Ca2 calcium ion, the neighboring G128 is twisted away from the H141, and the bond between G128 and H141 is broken, the distance and distance variance is increased, and thus the zinc environment destabilized further (Figure 3, right panel, in Text S1).

H183, the other zinc co-ordinating histidine, lies distal to the 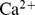 binding sites and is stabilized by a hydrogen bond with the carboxylate of E54, also distal to the 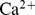 binding sites. Consequently, the geometry of this interaction is barely affected by calcium ion binding (Figure 3, left panel, in Text S1).

### Ca2 calcium ion switches the putative catalytic water

According to the enzymatic mechanism postulated by Hall *et al.* [9], there is another essential component of the catalytic zinc center: a water molecule occupying the fourth corner of the tetrahedron formed by the zinc ligands, as reported in the first X-ray structure [9]. This is another commonality with active LAS enzymes as noted by [10] (see Table 13 in Text S1). The accessibility of this co-ordination site to water is a necessary condition for an active enzyme with the same reaction mechanism, and it is fulfilled in ShhN.

**Figure 6:**
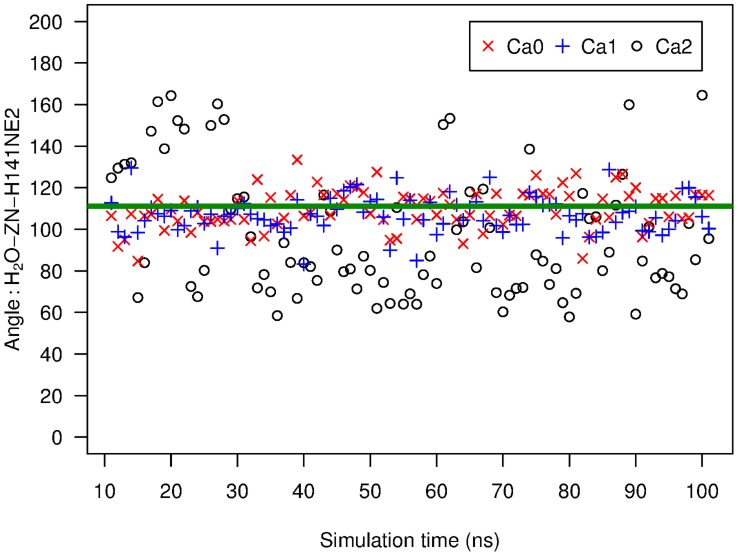
Angle between oxygen of putative catalytic water, zinc ion, and H141. Angles (in degrees) sampled by MD simulations of ShhN in states Ca0, Ca1, Ca2. Reference atom for angle measurements in zinc ligand H141 was 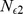. For comparison, the green line marks the angle in X-ray structure 1vhh [9].

If ShhN is a peptidase that employs the postulated mechanism, including the involvement of the water molecule at the zinc ion, and if further, this peptidase function is switched off by the Ca2 calcium ion, then we could expect that states Ca0 and Ca1 favor a water molecule at that position compared to Ca2. We have therefore analyzed our MD simulations for the behavior of water molecules close to the position of the putative catalytic water in terms of distance to the zinc ion and angles with other zinc ligands, e.g. the angle 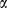 between 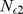 of H148, 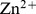, and water oxygen. There was in all simulations, irrespective of state (Ca0, Ca1, Ca2) almost always a water molecule at a distance of 0.2 nm to the zinc ion, i.e. at the distance of the putative catalytic water in X-ray structure 1vhh (Figure 5 in Text S1). This is not too surprising as the oxygen atom of water and the zinc have opposite partial charges, and therefore, given the water accessible zinc co-ordination site, there will always be a water molecule realizing this interaction. The angle 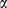 showed a more interesting pattern. We found for states Ca0 and Ca1 that a water molecule constantly has an 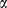 value very close to the crystallographically determined 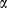 (Figure 6). In these states, this water molecule does not exchange with other water molecules during the simulations. The picture looks qualitatively different for state Ca2. There, the water molecule closest to the position of the putative catalytic water assumes clearly different positions, and also flips between distinct 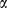 values (Figure 6). In Ca2, we could also observe exchanges of water molecules at the zinc ion. To completely define the position of the water molecule we have determined another angle (Figure 6 in Text S1), but the message remains the same, i.e. in Ca0 and Ca1 states, the water is fixed close to the experimentally observed position, while the water in Ca2 shows much more dynamics and in general is off the Ca0 crystal position. This loss of a well-defined water co-ordination also affects the flexibility and position of catalytic E177, linked to this water by a H-bond. This contributes to the widening and loosening of the H135-E177 clamp introduced above.

This behavior of the putative catalytic water molecule is consistent with the conformational switching of the zinc environment between more LAS-like in Ca0 and Ca1 to less LAS-like in Ca2. The conservation of a water molecule at this well-defined position in states Ca0 and Ca1 makes also sense in the light of a hydrolase function in these states, and the loss of this water co-ordination is in agreement with a loss of hydrolase function in state Ca2.

**Figure 7:**
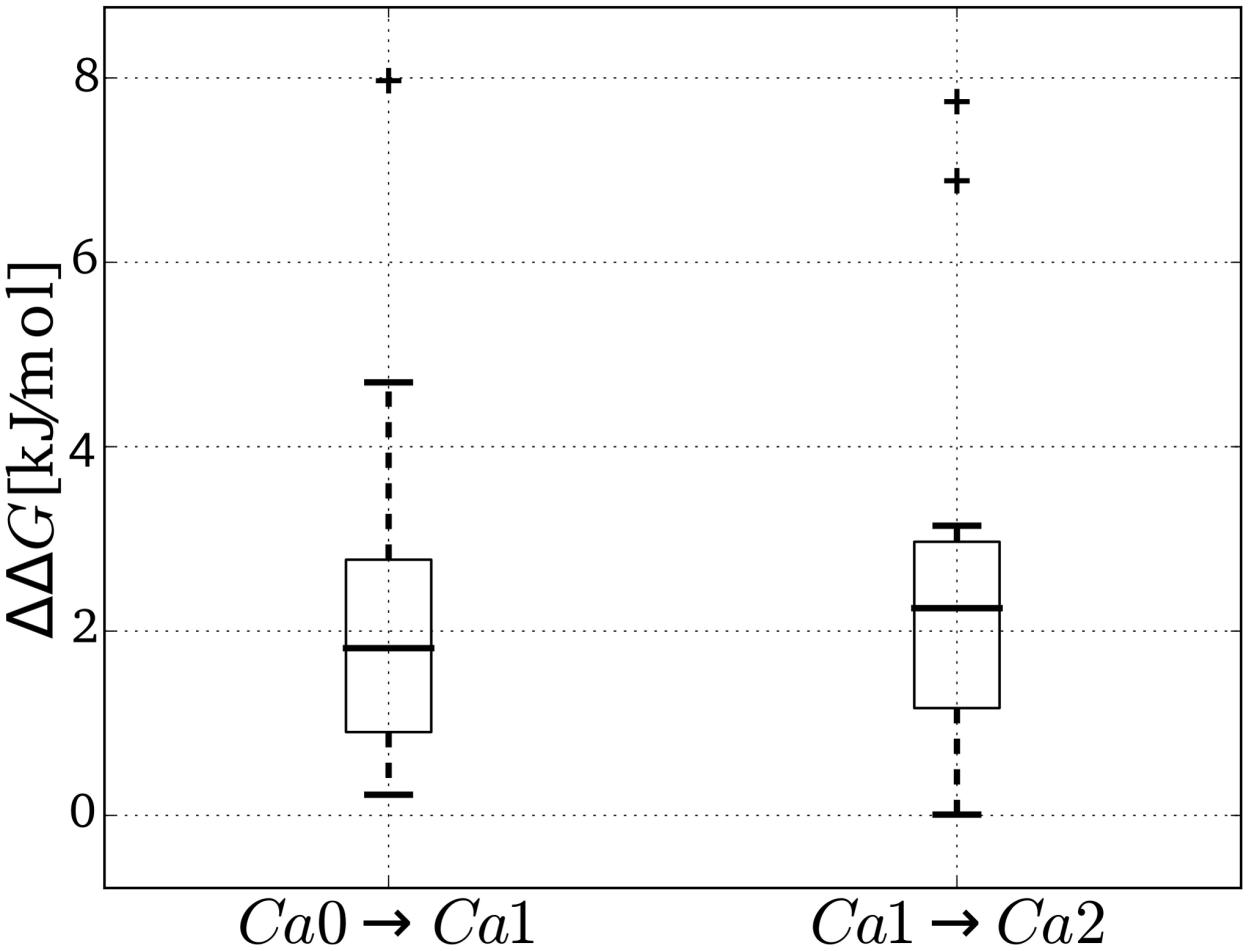
Electrostatics effect of calcium binding on affinity of catalytic water. Electrostatics based affinity differences 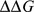 of the zinc co-ordinating water molecule between calcium binding states Ca0 and Ca1 (left), and between states Ca1 and Ca2 (right). Boxplots based on 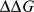 values computed for the nine available structures of ShhN (see Materials and Methods). Whiskers, box top, and box bottom mark quartiles of distribution, bold horizontal line indicates median, crosses are outliers. Note that both boxes are completely above the zero-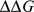 line, i.e. water binding is less and less electrostatically favorable from Ca0 to Ca1, and from Ca1 to Ca2.

The observed switch from the conservation of the zinc co-ordinating water molecule in states Ca0 and Ca1, to the more liquid-like behavior of water around the zinc ion in Ca2 could be solely due to conformational changes induced by the binding of the Ca2 calcium ion (opening of clamp), or there could be also contributions by direct electrostatic interactions between the calcium ion and the zinc co-ordinating water molecule. To test whether there is a noticeable direct electrostatic interaction, we solved the Poisson-Boltzmann equation for different calcium states of ShhN, (a) in complex with the zinc co-ordinating water, and (b) with that water released into the bulk water. The corresponding affinity differences 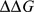 of the zinc co-ordinating water to ShhN were computed for different ShhN structures. Figure 7 shows that for each added calcium ion the electrostatic energy of the water molecule increases, i.e. the affinity of the water molecule is diminished; this is true for all ShhN structures. Thus the behavior of the water molecule as a function of calcium state observed in the molecular dynamics simulation can be partially attributed to direct electrostatic interactions between the 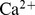 ions and the dipole of the zinc co-ordinating water.

### Do non-enzymes have zinc centers similar to ShhN?

While the evidence presented so far is compatible with an enzymatic function of ShhN, it is unclear whether the structure of the zinc center is specific for enzymes, or whether other non-enzymes show a similar structure. To test this, we have searched with EpitopeMatch [27] the complete PDB (last access Jan 30, 2013; see Methods) for an arrangement similar to the one in the putative active site of ShhN [9], including the following eight groups: zinc ion, E127, H135, H141, D148, E177, H181, H183 (PDB entry 1vhh). Not surprisingly, all 20 X-ray structures of Hedgehogs were found with complete coverage of all eight groups and low RMSD (0 - 0.1 nm). Further, we found two matching proteins covering seven of the groups, a phosphodiesterase (1bf6) and a putative metalloprotease (3iuu). When we allowed for conservative exchanges of amino acids, another group of proteins were found also with full coverage of eight residues, all of them enzymes, including eight LAS enzymes (PDB entries 1lbu, 1qwy, 1r44, 1u10, 2b13, 2b44, 2vo9, 4f78), a hydrolase (3csq), and Thermolysin (8tln). No other proteins with a similar center were found. Thus in the set of available protein structures, the zinc center in ShhN was characteristic of enzymes.

### Evolutionary conservation

**Figure 8:**
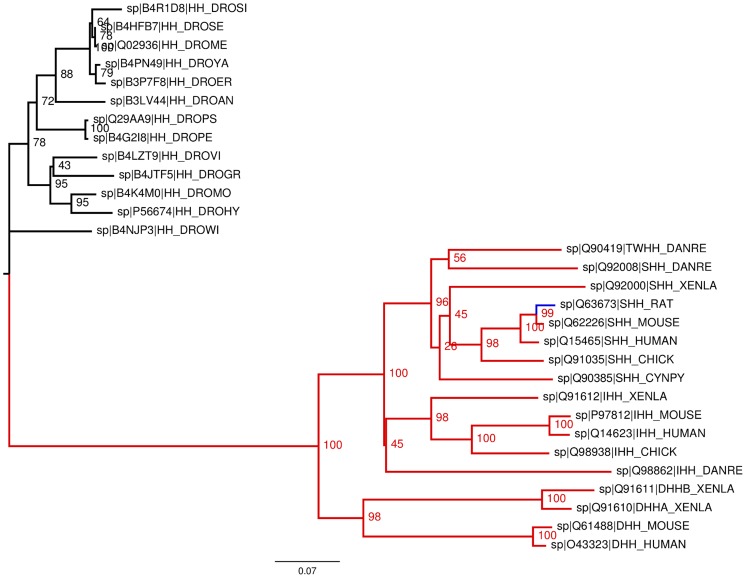
Phylogenetic tree of all 30 reviewed full length Hedgehog proteins from UniProtKB. The vertebrate Hedgehogs (bottom subtree) are clearly separated from the *Drosophila* Hedgehogs (top subtree). In all vertebrates the full catalytic motif is absolutely conserved (red), except in rat with one conservative exchange (blue).

Having an enzymatic function in an organism or not having it seems to be a fundamental property of a protein. Accordingly, one intuitively expects that the putative catalytic center of ShhN is evolutionary conserved. However, it had been noted from early on that in *Drosophila* the zinc binding site is not conserved [9], [28]. To assess the degree of evolutionary conservation we therefore computed a phylogenetic tree of Hedgehog proteins (see Methods) and checked where on the tree the putative catalytic center is present (Figure 8). The gross topology of the tree agrees with that of earlier trees [29] based on fewer sequences, and shows a clear branching between *Drosophila* hedgehog proteins and vertebrate hedgehog proteins, including Sonic, Indian, Desert, and Tiggy-Winkle hedgehog. We found that 16 out of 17 vertebrate hedgehog sequences contain the full putative EHHDEHH catalytic motif corresponding to mouse ShhN E127, H135, H141, D148, E177, H181, H183, while all of the *Drosophila* sequences carried instead the motif EHHTVHY, with the D148/H183 co-ordinating zinc in ShhN replaced by a threonine/tyrosine in *Drosophila* hedgehog. The only sequence in the vertebrate branch that has not the full active site motif is ShhN of rat. In this sequence, all zinc co-ordinating residues are present, but H181 is replaced by an arginine. H181 is not co-ordinating the zinc ion but may fixate the catalytically active E177 in a position appropriate for interaction with the catalytic water. To test this, we returned to the structural comparison of the ShhN zinc center with the zinc centers of the LAS enzymes, and we found that there is at least one confirmed LAS enzyme, L-alanoyl-D-glutamate endopeptidase of a bacteriophage (PDB entry 2vo9; [30]) that has a proline at the structural position corresponding to H181. This means that a histidine at this position is not strictly required for peptidase function, and hence rat ShhN could still be a peptidase. These results are compatible with a conservation of ShhN enzyme function in vertebrates. In the insect class the picture is less clear: while *Drosophila* hedgehog has no zinc center, Mosquito hedgehog has all seven residues of the putative ShhN active site [9].

Surprisingly, the carboxylate carrying residues (in mouse ShhN E90, E91, D96, E127, D130, D132) that co-ordinate the calcium ions are almost completely conserved in all 30 sequences, including *Drosophila* and vertebrates. The only exception is *Drosophila ananassae* Hedgehog protein in which E90 is conservatively replaced by an aspartate.

### Peptidase substrate

If ShhN is an enzyme, there should be a substrate. So far, no substrates of ShhN have been described, despite efforts to identify such substances [9], [16]. We tried to narrow the set of possible substrates by using the available ShhN structures. In our search we restricted ourselves to peptide substrates, as most of the proteins with the highest structural similarity to the putative ShhN catalytic center are peptidases or peptidoglycan amidases [10].

Hedgehog Interacting Protein (Hhip) is a functionally important binding partner at the zinc centers of ShhN and the corresponding DhhN domain in the related Desert Hedgehog protein [12]. In X-ray structures of Hhip – ShhN complexes, Hhip binds with a negatively charged patch to the zinc center. Hhip affinity to ShhN and DhhN is influenced by the calcium concentration: for high 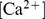, the 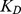 for binding of Hhip to ShhN and DhhN is on the order of 10 nM, for low 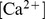 of the order of about 100 nM [12]. This means that under the low 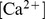 conditions hypothesized to trigger the peptidase function, the affinity of Hhip to ShhN is lower. Moreover, Hhip replaces the putative catalytic water molecule by the carboxylate group of a glutamate side chain, similar to the binding of inhibitor TIMP-1 to its cognate zinc protease MMP-3 [13], [15]. Thus, Hhip is probably not a good substrate model.

**Figure 9:**
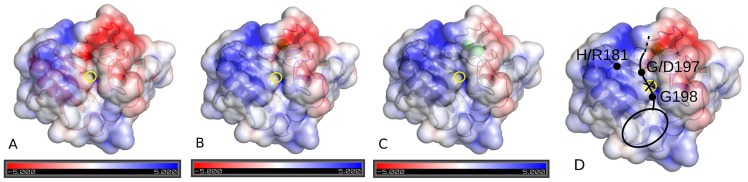
Electrostatic potential on solvent accessible surface (SAS) of ShhN in three calcium binding states. Panels show Ca0 (A), Ca1 (B), Ca2 (C), and model of ShhN-substrate complex (D) (PDB entry 3d1m). SAS is transparent to allow view on zinc (highlighted by yellow circle) and calcium ions (larger green spheres in upper right part of molecule). Electrostatic potential 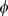 coded by color and given on SAS. 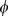 is given in units of 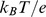, and a value of 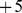 corresponds to about 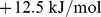 for singly charged molecules at room temperature. View is directly on putative catalytic center around zinc ion. Panel (D) shows Ca1 based model of the complex of ShhN peptidase with its substrate (black lines), i.e. the C-terminus of a neighboring ShhN, including the cholesterol modification (ellipse). Potential cleavage position is indicated by cross at zinc center. Potential substrate is embedded into hydrophobic pocket (cholesterol) and crevice (peptide G197, G198). Note proximity of H/R181 and G/D197 (compensatory mutations in rat).

The authors of the 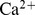 free ShhN X-ray structure [9] (PDB entry 1vhh) noted that in the crystal, one ShhN molecule binds to the C-terminal peptide of its crystal lattice neighbor, so that this peptide comes close to the zinc ion but without replacing the putative catalytic water. The C-terminal carboxylate oxygen forms an isosceles triangle of side length 3.4 Å with the 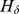 of H135 and the zinc ion, but is only 2.5 Å away from the oxygen of the catalytic water. It is well imaginable that this situation represents the state of the peptidase before the dissociation of the product of the cleavage reaction. Except for the last amino acid, the C-terminal peptide SVAAK in 1vhh is not particularly polar. The amino group of the terminal lysine side chain is not engaged in contacts with the protein, but points away from it. This means that the putative active site binds non-polar peptides, and thus that the peptide substrate could be non-polar. If this is true, we should see for the putative enzymatically active states Ca0 and Ca1 a low electrostatic potential close to that binding site, while the potential should flip to higher values in Ca2 to allow for binding of Hhip. To test this we have taken a high resolution X-ray structure of Ca2 state (PDB: 3d1m) and computed the electrostatic potential around ShhN in Ca0, Ca1, Ca2 states, with the former two generated by removing calcium ions from the structure. Figure 9 shows that the zinc center region indeed changes electrostatic potential from slightly negative (Ca0), over slightly positive (Ca1), to high (Ca2). This means that in the putative enzymatically active states Ca0 and Ca1 the zinc center region has an electrostatic potential close to zero, suggesting non-polar substrates, similar to the C-terminal peptide of ShhN discussed above.

In functional vertebrate ShhN, the C-terminal peptide is not SVAAK as in the X-ray structure 1vhh, but SVAAK(S/T)GG, which is still compatible with a rather non-polar peptide substrate. The only known exception to this rule in vertebrates is again rat where the first G (G197) is replaced by D197. Recall that in rat we also have mutation H181R. Hence, two neutral residues, H181 and G197, are replaced by two oppositely charged residues, R and D. This could be a compensatory pair of mutations if both residues were close in space. In fact, 1vhh shows that, if we would extend the C-terminus by (S/T)(G/D)G, the G/D197 in the C-terminal peptide of one ShhN protein could be very close to H/R181 of a neighboring ShhN protein (Figure 9D). Such a compensatory mutation makes sense, if the proximity between zinc center and C-terminal peptide observed in Ca0 (1vhh) is functionally relevant, e.g. if the C-terminal peptide is a substrate of a ShhN peptidase.

As mentioned above, ShhN proteins form large oligomers [4], a situation in which many ShhN molecules are close neighbors, and thus may expose their C-termini or other hydrophobic parts to cannibalysis [18] by neighboring ShhN molecules.

### Conserved glycines in ShhN as possible cleavage sites

Amongst the LAS enzymes, the lysostaphins have zinc centers that are most similar to ShhN. Lysostaphins are known to have slight elastase activity [31], and most of lysostaphin-like enzymes are believed to cleave glycyl-glycine or glycyl-alanine peptide bonds [10]. Hence, conserved glycines in the lysostaphin-like ShhN could be potential cleavage sites for an autodegrading ShhN peptidase.

There is only one glycyl-glycine motif in ShhN, right at the C-terminus of ShhN. This GG motif is conserved in vertebrates, i.e. in organisms with an intact zinc center, but not in *Drosophila*, where the zinc center is missing. Cleavage of the peptide bond between these glycines would be functionally relevant, as the C-terminal G198 carries an important cholesterol modification [32]–[34], and cleavage of the C-terminal glycine would remove this cholesterol. At the molecular level, removal of cholesterol may e.g. dissolve ShhN oligomers [35] and thus expose ShhN to other peptidases, or it could increase the mobility of ShhN [36]. Very recently, it has been reported that a fraction of ShhN may act in an unlipidated form, though it remained unclear how this form is generated [37]. The removal mechanism of the cholesterol by ShhN proposed here could contribute to this fraction.

A GG-cholesterol motif is also a noteworthy substrate candidate because of its hydrophobic nature. Remarkably, there is a sizable, shallow hydrophobic pocket close to the zinc (black ellipse in Figure 9D), extending from L140 over W173 to F48. This pocket has an electrostatic potential close to zero, independently of the 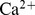 binding state (Figure 9), which means that the binding of the cholesterol group could be decoupled from the peptidase activity.

To test whether the shallow hydrophobic pocket is suitable for binding cholesterol, we have applied molecular docking to the complete surface of ShhN as receptor and cholesterol as ligand. As the proposed binding pocket lies in the vicinity of the zinc ion, and the zinc is presumed to be co-ordinated in states Ca0 and Ca1 by a well-defined water molecule, we carried out two docking runs, one with this water and one without it. In both runs the hydrophobic pocket close to the zinc was identified as the dominant binding site, accommodating the cholesterol molecule in various orientations and poses (Figure 4 in Text S1). There was only one further binding site in a distance of more than 2 nm to that pocket, but this alternative binding site turned up at rank 14 or worse amongst the 20 best poses (Tables 10 and 11 in Text S1).

The binding of the cholesterol moïety in this hydrophobic pocket is compatible with the observed pair of assumed compensatory mutations between mouse (H181, G197) and rat (R181, D197): If we place G/D197 close to H/R181, the C-terminal G198 will hover just above the zinc ion, pointing with its cholesterylated C-terminus to the hydrophobic pocket (Figure 9D).

This extended hydrophobic pocket also indicates a possible mechanism for the extraction of cholesterol from the membrane. The pocket is large, shallow, and well-accessible at the surface of ShhN. Thus, ShhN may dip with this part into the membrane and recruit a cholesterol C-terminally attached to a neighboring ShhN. Similar mechanisms have been proposed for other lipid binding proteins with extended hydrophobic regions [38], [39].

The other substrate pattern suggested by comparison with lysostaphins is glycyl-alanine. There are two such GA motifs conserved in ShhN. The first lies at G58, and this GA motif is conserved in vertebrates, but not in *Drosophila*. The other lies at G94 and is almost conserved in all Hh proteins (including *Drosophila*), except for Tiggy-Winkle Hh in zebrafish where this G is replaced by N. The GA motif at G58 seems to be a better candidate as the glycine is well-accessible, and it is part of a predominantly hydrophobic peptide TLGASG that is strictly conserved in all vertebrate Hh, but not in *Drosophila*.

The set of potential cleavage sites discussed above could be extended to a total of 13 conserved glycines, several of which are well exposed at the surface and have hydrophobic neighbor residues.

### *In vitro* experiments

So far, the presented arguments for the predicted calcium-regulated autodegrading ShhN peptidase have been based mainly on simulation results and on comparisons with enzyme structures, but no direct experimental evidence for this hypothesis has been available. In early tests, no ShhN peptidase activity could be detected with peptide substrates [9], [16], including peptide stretches from ShhN. It had been shown for other peptidases that the structural presentation of the peptidase substrate can be relevant [40]. Moreover, earlier tests were based on bacterial expression systems that lack the capability of adding functionally important modifications to ShhN, such as the N-terminal palmitoyl and the C-terminal cholesterol. We therefore asked whether a potential autodegrading peptidase function of ShhN may primarily target natively folded and properly modified ShhN.

For a first test, we wanted to establish a mutant ShhN (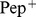) that by construction is in state Ca0 and therefore should be a constitutively active peptidase. To this end we introduced mutations E90A, E91A, E127A, expected to weaken the affinities in both calcium binding pockets (Figure 2B). In MD simulations, 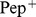 did not show stable calcium binding. In the putative catalytic center, the structures sampled by the MD simulations of 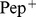 had an RMSD that was significantly lower to the Ca0 structure 1vhh than to the Ca2 structure 3d1m (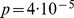).

Further, we constructed a four-fold mutant (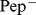), carrying the three mutations of 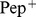, but additionally mutation E177A, which eliminates the residue assumed to play the central catalytic role. Thus, 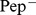 should not be a peptidase.

**Figure 10:**
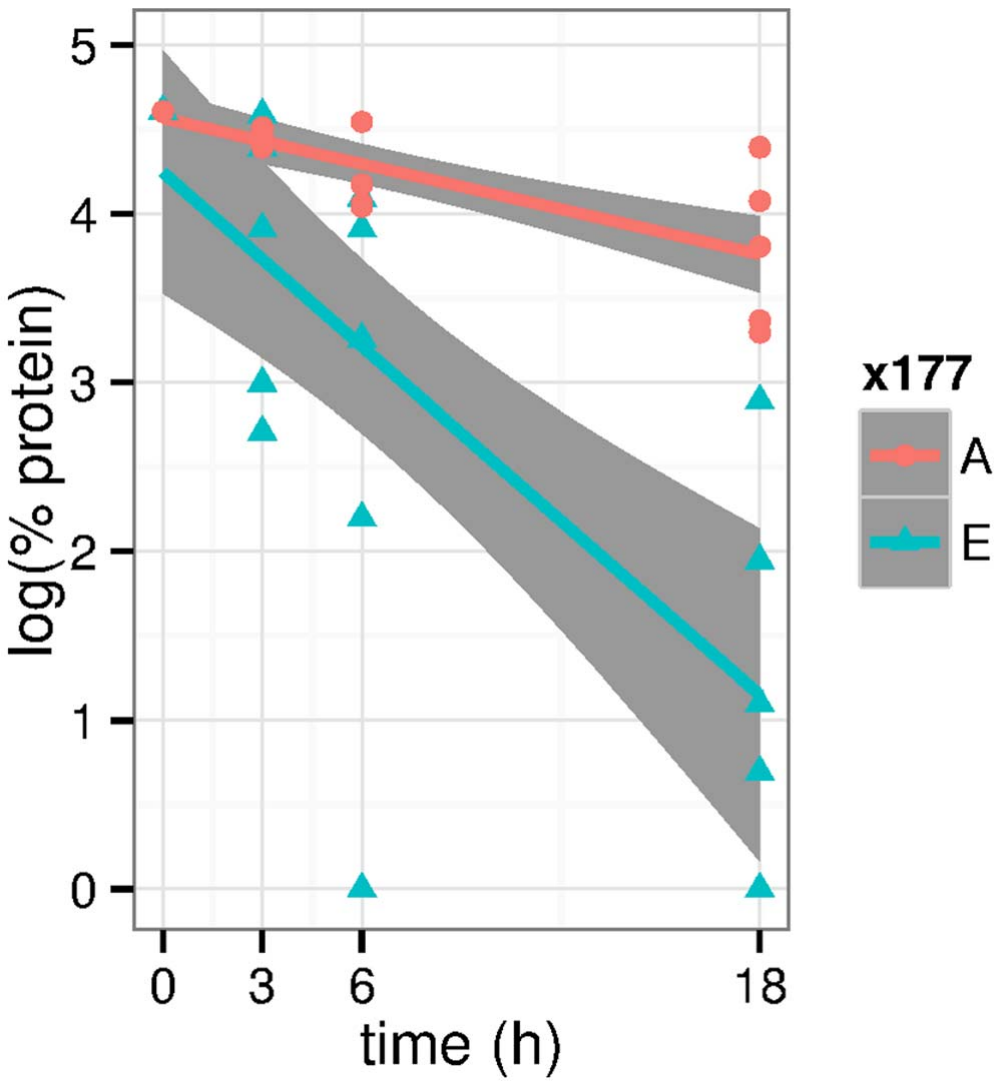
*In vitro* tests of ShhN mutant stabilities against proteolysis. The logarithm of protein content (relative to maximum protein content) is plotted over time. Proteins are 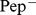 (E177A, red) and 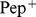 (E177, blue). Straight red and blue lines are least squares fits to the measurements, shaded areas around these lines are 95% confidence intervals for the corresponding linear models. All data refer to measurements at 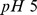.

If the mutations in 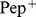 did not abolish the cleavage site, we expect that a self-degrading 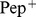 peptidase is unstable in an autodegradation assay while 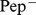 should be stable. We tested this *in vitro* (data in Table 14 in ). We found that at 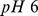 both ShhN mutants were stable for many hours (Table 14 and Figure 9 in Text S1). At 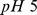, both mutants were degraded (Figure 10). However, the decay of 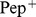 was much faster leading to significantly lower protein content at 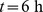 (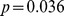 in Wilcoxon test) and 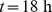 (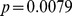). At the latter time point, 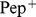 had almost completely vanished, while there was still about half of the 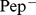. A least squares fit to an exponential decay (straight lines in Figure 10) yields a half-life of 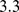 to 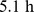 for 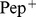 and of 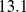 to 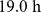 for 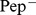 with the boundaries computed from fitted slopes 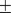 standard errors.

The fact that not only 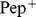 but also 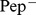 decayed at 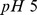 can be attributed to other proteases in the medium that cannot be completely eliminated in this genetic experiment and that are active at this 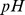. However, the highly significant difference between 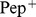 and 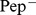 can be most easily explained as difference between an autodegrading peptidase 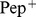 and a knock-out of the catalytic function in 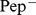 by point mutation E177A.

Alternatively, we could explain the result if we assume that neither 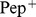 nor 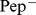 is a peptidase and that E177A abolishes a recognition site for another peptidase that is present in the medium. Although we currently cannot rule out this more complex hypothesis, inspection of the available structures shows that E177 lies protected in the putative substrate binding crevice of the ShhN peptidase, and it therefore seems to be not plausible that this is a recognition site for another protease.

### 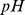 dependency of ShhN stability

Peptidases often have a 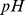 range of optimal activity [41]. Thus, it is not surprising that the stability of ShhN in the above *in vitro* experiments had a strong dependence on 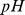, with a decay of ShhN at 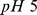 and stable ShhN at 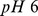. A simple interpretation of this observation is that the ShhN peptidase is more active at 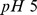 than at 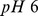. We therefore estimated 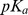 values for all protonatable groups in ShhN based on structure 1vhh [9].

We found that the protonatable groups in the calcium binding site have 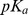 values of 6 and lower (Table 12 in Text S1). This means that at neutral 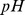 the binding site should be fully deprotonated. However, there are in the calcium binding site three carboxylate groups (E91, D96, E127) with 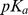 between 5 and 6, indicating a tendency for protonation at 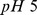 and thus a weaker affinity to 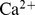. Thus, ShhN peptidase could be switched *on* by shifting the 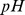 from neutral to 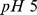. For the observed difference between 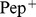 and 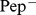, this additional switch is irrelevant since both mutants do not bind 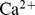. There is only one further group with a 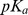 between 5 and 6, namely H181, with a 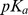 of 5.5 in Ca0 (1vhh). In the first X-ray structure [9], H181 is described as forming a charged hydrogen bond with the carboxylate of putative catalytic E177, and thus stabilizing the latter. According to our 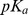 estimate, the shifting of 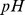 from 5 to 6 would make protonation of H181 less likely and thus destabilize E177. This could in part explain the observed 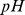 dependency of 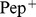 stability in our experiments (Figure 9 in Text S1).

## Conclusions

The presence of a calcium-regulated zinc peptidase function of ShhN would explain the following observations:

1. The close similarity of the ShhN zinc center to the the centers of LAS enzymes. Apart from ShhN, all proteins with such a center are enzymes, mostly peptidases.

2. According to MD simulations and X-ray structures, the loss of calcium ions makes the ShhN zinc center significantly more similar to the zinc center of LAS enzymes.

3. In states Ca0 and Ca1, a water molecule stably co-ordinates the zinc ion, in position and pose matching catalytic waters in related enzymes. This water position and pose is disfavored in state Ca2.

4. The outcome of our comparative genetic experiments with mutants 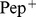 and 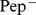: If our hypothesis is correct that calcium loss activates the peptidase function and that E177 is the key catalytic residue, 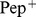 should be active, 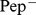 inactive, which is compatible with our observation.

While other explanations for these observations are imaginable, the presence of a calcium regulated zinc peptidase function in ShhN seems to be the most parsimonious one. Early attempts to find peptidase function relied on bacterially expressed ShhN that is lacking functionally relevant modifications, as outlined above. These modifications are important for the formation of ShhN oligomers [5], and we have argued above that oligomerization and autodegradation could well be coupled.

In summary, we find that our computational and experimental results are in agreement with the hypothesis of ShhN as a calcium regulated autodegrading zinc peptidase. According to our model, the peptidase function should be switched off if both calcium binding pockets are occupied. The switching mechanism involves dragging of glutamic acid residue 127 towards the calcium center, a movement that is transmitted to the zinc center through several residues coupled to E127, notably histidine 135 which is bound to E127 by a hydrogen bond. The dragging of E127 pulls this H135 away from the putative substrate. Another notable effect of the binding of the second calcium is the destabilization of a catalytic water molecule that co-ordinates the zinc ion.

These conformational changes typically amount to distance changes of about 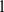 to 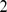 Å. Yet, such small conformational changes can leverage hundred to thousandfold changes of enzymatic activity (Ref. [42], p. 166). The propagation of functional conformational changes of this size through proteins has been observed before [43].

While our *in vitro* experiments are consistent with the hypothesis that ShhN is a calcium regulated autodegrading peptidase, the target motifs of this peptidase have still to be determined. By comparison with Lysostaphins we have proposed conserved GG or GA motifs as targets, e.g. the C-terminal GG-Cholesterol motif, or the GA motif at G58 that both are conserved in vertebrates, but not in *Drosophila* where no zinc peptidase activity is expected. The importance of these motifs for autodegradation could be tested by site-directed mutagenesis and mass spectrometry experiments.

Our discussion of the two mutations in rat ShhN compared to mouse ShhN has led us to conclude that we have a pair of compensatory mutations: 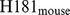 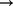 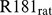, and 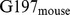 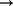 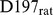, with the latter a direct neighbor of the C-terminal G198. Accordingly, we predict that chimeric ShhN with combinations H181/D197 or R181/G197 should be partially compromised in their peptidase function.

The predictions above are experimentally testable. It is also clear that autodegradation could be an elegant mechanism to tune morphogen gradients [6], [7]. It is currently less clear where and when *in vivo* the calcium regulation of peptidase function comes into play, though phylogenetic conservation of all parts of its mechanism supports its relevance.


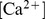
 in blood is usually tightly controlled at a relatively high level, and hence this does not seem a likely place for enzymatic activation. More promising are interstitial or intracellular compartments. In various interstitial fluids 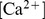 values between 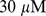 [44] and 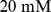 [45] have been reported, so that there the hypothesized enzymatic function could be switched on and off. In neural tissue, 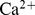 concentrations can be transiently depleted [46], [47]. ShhN could also be internalized into cells and exposed to lower 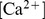 there. Finally, it is conceivable that ShhN competes for calcium ions with the negatively charged proteoglycans of the extracellular matrix, and in the course of this competition temporarily loses calcium ions and gains peptidase function. If this is true, we should find that autodegradation depends on the proteoglycan composition in the extracellular space.

The *in vitro* experiments have pointed to a strong 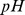 dependence of protease activity with the peptidase being inactive at 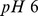 and active at 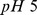. This raises again the question where and when such acidic conditions may be fulfilled. There are acidic intracellular compartments (lysosome or late endosome), but a more interesting candidate in view of the mode of action of Hhs is again the extracellular matrix which abounds with proteoglycans rich in acidic groups that lower local 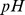. In fact, it is well-known that proteoglycan - Hh interactions are important for Hh function [48], [49].

The autocleavage of a terminal peptide at low 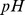 is a feature that ShhN could share with some zymogens such as pepsinogen, thus ShhN could be an autoactivating and autodegrading zymogen tuning its own concentration gradient. This does not imply that this is the only mechanism that shapes the ShhN concentration gradient. For instance, sheddases have been shown to contribute to ShhN function [35].

Although the work presented here is consistent with the hypothesis that ShhN is an autodegrading peptidase that is switched off by the binding of the Ca2 calcium ion, this does not preclude other functions of this calcium ion. It had been shown experimentally that CDO, a mammalian receptor of ShhN, binds ShhN preferentially in Ca2 state [19]. CDO does not bind at the putative substrate binding site, but closer to the 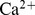 binding pocket. The electrostatics of this region depends heavily on the presence of calcium, as we could show by computing the electrostatic potential of ShhN structures in states Ca0, Ca1, and Ca2 (Figure 7 in Text S1). On the other hand, it is remarkable that in *Drosophila*, one of the few known cases where Hedgehog has no zinc center, the CDO analog Ihog binds to a different region of Hedgehog, independently of calcium [19]. This could indicate that at the level of the molecular interaction network, both functions, CDO/Ihog binding and ShhN peptidase activity, may be linked.

## Materials and Methods

### Molecular structures

All molecular structures were retrieved from the Protein Data Bank PDB [50]. Three different states – here called Ca0, Ca1, Ca2 – of murine ShhN with respect to 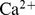 binding were studied: ShhN without calcium (Ca0) based on PDB entry 1vhh [9], ShhN with one 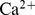 (Ca1) based on PDB entry 3n1r [26], and ShhN with two 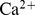 (Ca2) based on PDB entries 2wfx [12] and 3d1m [19]. In the latter two structures, ShhN is bound to Hhip and Ihog, respectively, and if necessary we therefore refer to these structures as 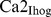 and 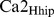. For consistent comparison, all structures of Ca0-Ca2 were considered between residues L40 and E189, a range common to all structures. As the few clipped residues were disordered and far from the metal binding sites, this manipulation was not considered critical.

ShhN structures were compared to structures of five representative LAS enzymes: *Streptomyces albus G* D-Ala-A-Ala Carboxypeptidase (PDB entry 1lbu; [51]), L-alanoyl-D-glutamate endopeptidase (PDB entry 2vo9; [30]), VanX amino-peptidase (PDB entry 1r44; [11]), and peptidoglycan amidase MepA (PDB entry 1u10; [52]).

### Molecular dynamics simulations

The molecular dynamics of ShhN, ShhN mutants (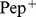, 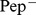), and LAS enzymes was simulated with the GROMACS 4.6.5 package [53]. Nonbonded interactions were calculated on a GPU (GeForce GTX 780), and bonded interactions and PME summation on four CPUs. The GROMOS96 43a1 force field was used with optimized Lennard-Jones parameters 
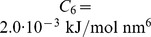
 and 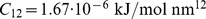 for 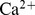 interactions taken from Ref. [54]. For water we used the SPC/E model, as recommended for simulations with GROMOS96 force fields in the Gromacs documentation.

Each system was simulated in duplicate using the following protocol. Initial protein structures were solvated in a rhombic dodecahedron box of SPC/E water with a minimum of 1.0 nm distance between protein and faces of box. Residues were assumed to be protonated according to their normal states at 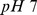, with the exception of histidines. The protons were assigned to histidines after inspection of H-bond patterns in ShhN X-ray structures to the following nitrogens: 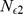 for H134, H181, H183; 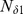 for H141, and both for H135. 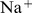 and 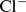 ions were added to neutralize the system at an ionic strength of 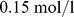. The Particle Mesh Ewald method was used to compute electrostatic interactions under periodic boundary conditions. Structures were energy minimized and equilibrated by Molecular Dynamics simulation for 5 ns. Production simulations were run for 100 ns with a time step of 2 fs. NPT conditions were stabilized at 300 K by V-rescale thermostat, and at 1 atm by Parinello-Rahman barostat. Bonds were constrained using the LINCS algorithm. Snapshots of the trajectories were saved every 100 ps. The last 90 ns of all trajectories did not show increasing root-mean-square-deviations (RMSDs) with respect to the starting structures, but merely fluctuations. We analyzed the trajectories with the toolbox provided by GROMACS, especially g_rms for RMSDs and g_rmsf for root-mean-square-fluctuations (RMSFs). Representative structures for the different calcium binding states in Figure 2 were extracted from trajectories with g_cluster based on mutual RMSDs; the structures shown are close to the cluster centers and represent about 90% of the trajectories. Structures in trajectories were matched with other structures using EpitopeMatch [27] (see next section). For all statistical analyses we used R version 3.01 [55].

### Matching of molecular structures

Similarity of molecular structures was quantified with EpitopeMatch [27], version 2013.09.20 (see also http://www.EpitopeMatch.org). For two molecular structures or structures of discontinuous fragments given by their geometries (e.g. atomic coordinates) and physico-chemical properties (e.g. atom types), EpitopeMatch aligns the two structures so that the Euclidean distances between physico-chemically similar parts are minimized. The quality of the match between the two structures can be described in terms of root-mean-square-deviation (RMSD), number of matched components, or a similarity index that combines several quantities. Here we have used a matching mode of EpitopeMatch in which the two structures are aligned so that a maximum number of chemically identical atoms from both structures is superimposed, and between these the match with the lowest RMSD is selected.

EpitopeMatch was used here for three purposes: (1) to characterize similarity between LAS enzyme zinc centers taken from X-ray structures and ShhN structures taken from MD trajectories; (2) to characterize the similarity between LAS enzyme zinc centers based on their MD trajectories; (3) to identify in the complete PDB (about 88.741 structures) arrangements similar to the zinc center of ShhN.

### Estimation of calcium ion affinities

We have used an empirical model to compute rough estimates of 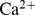 affinities to the two binding sites [25]. Basically, the model correlates Gibb's free enthalpy of binding 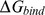 with the size of the solvent accessible surface of the 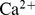: the more exposed the ion, the weaker the affinity. Quantitatively, we estimate
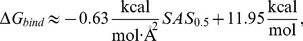
(1)with 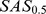 the accessible surface for a probe sphere radius of 0.5 Å. This formula was applied to both calcium ions in the three ShhN X-ray structures in state Ca2 with PDB entries 3d1m, 3mxw, 2wfx. The first approximations of the affinities thus obtained are 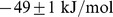 for Ca1 and 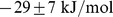 for Ca2 (errors are standard deviations from calculations on the different X-ray structures). However, these absolute values are overestimating the affinities as the empirical model does not consider interactions between the closely neighboring calcium ions, and the model does also not account for the loss of entropy due to conformational condensation, especially on binding of the first calcium ion (Figure 1). We therefore use only the difference of the above estimates, 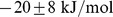, as an rough estimate of 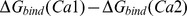, indicating that Ca1 is bound more tightly than Ca2.

### Electrostatic interactions between zinc co-ordinating water and calcium ions

Electrostatic potentials were computed by solving the non-linear Poisson-Boltzmann Equation with APBS [56] using a grid with 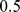 Å spacing, dielectric constants of 79 and 2 outside and inside ShhN, respectively, and a water probe of radius 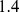 Å. Temperature was set to 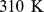, ionic strength to 0.1 mol/l NaCl outside ShhN and 0 mol/l inside ShhN. Charges and radii were assigned with PDB2PQR Version 1.7 [57] using the AMBER99 [58] force field parameters.

We retrieved ShhN structures 1vhh, 2wfq, 2wfx, 2wg4, 3d1m, 3ho5, 3m1n, 3mxw and 3n1r from the PDB. After structural alignment of the zinc ligands, we used the catalytic water coordinates from the PDB entry 1vhh and the calcium coordinates from PDB entry 2wfx for the setup of the three states Ca0, Ca1, and Ca2. Protonations of histidines were consistent with those used for the molecular dynamics simulations.

For each structure and its three states we calculated the electrostatic component of the binding free energy 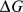 between catalytic water and protein using a standard thermodynamic cycle. The differences 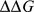 of electrostatic binding free energies between the states (Figure 7) was then calculated as:
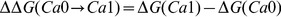
(2)

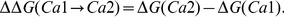
(3)


### 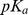 calculations

PROPKA version 3.1 [59] was used to estimate the 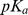 values of side chains in ShhN. Default parameters were used to perform the calculations. Coupling effects between protonatable groups were included.

### Phylogeny

From UniProtKB (last access Feb 30, 2013) we retrieved all 30 reviewed amino acid sequences of full length Hedgehog proteins. The set contained sequences of Desert Hedgehog, Indian Hedgehog, Sonic Hedgehog, and Tiggy-Winkle Hedgehog from *Drosophila* species and several vertebrates. The sequences were aligned with t-coffee [60], Version 8.99, with default settings. Based on this alignment we computed a distance based tree with BioNJ [61]. Branching confidence was assessed by 100 bootstraps.

### Docking

The cholesterol molecule was taken from ZINC database [62] (ID: 3869467). The mol2 file was translated to PDB format using Chimera v.1.6.2 [63]. The protein structure used was PDB entry 1vhh. The docking was prepared with AutoDockTools/MGLTools v.1.5.6 (The Scripps Research Institute) by adding polar hydrogens and assigning charges to all atoms. AutoDock Vina 1.1.2 [64] was used for all docking runs. General docking parameters for both calculations were kept at their default values, except for the exhaustiveness value, which was increased to 12. The docking grid had a size of 58 Å 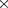 52 Å 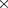 52 Å and covered the entire ShhN protein.

### Cloning and expression of recombinant proteins

Shh and Hh acyltransferase (Hhat) coding sequences were generated from murine cDNA (NM_009170) and human cDNA (NM_018194) by PCR. PCR products (Shh nucleotides 1-1314, corresponding to amino acids 1-438 of ShhN and Hhat nucleotides 1-1481, corresponding to amino acids 1-493) were ligated into pDrive (Qiagen, Hilden, Germany) and sequenced. 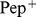 mutant lacking 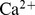-coordinating amino acids E90, E91 and E127 (mouse Shh nomenclature) was generated by site directed mutagenesis (Stratagene, La Jolla, USA). 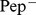 mutant, additionally lacking putative catalytic residue E177, was also generated by site directed mutagenesis. For primer sequences see Table 15 in Text S1. Both cDNAs, together with Hhat were cloned into pIRES (ClonTech, Mountain View, USA) for bicistronic ShhNp/Hhat co-expression in Bosc23 cells, a HEK293 derivate. This resulted in the secretion and proteolytic processing of N-palmitoylated and C-cholesterylated ShhNp termini, respectively.

### Cell culture and protein analysis

Bosc23 cells were cultured in DMEM (PAA, Cölbe, Germany) with 10% fetal calf serum (FCS) and 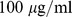 penicillin/streptomycin and transfected using PolyFect (Qiagen). After cells had been cultured for 24 hours, media were removed, cells washed with 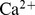-free PBS, and proteins secreted into serum-free/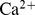-free EpiLife media (Gibco, USA) for 16 h. Media were harvested, ultracentrifuged, the 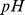 adjusted to 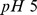 and 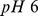, and incubated at 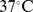 for 3 h, 6 h, and 18 h before Trichloroacetic acid (TCA)-precipitation. All proteins were analyzed by 15% SDS-PAGE, followed by Western blotting using PVDF-membranes. Ponceau-S staining of PVDF-membranes after blotting or Coomassie-staining of gels was conducted as loading controls. Blotted proteins were detected by polyclonal 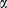-Shh antibodies (goat IgG; R&D Systems, Minneapolis, USA). Incubation with peroxidase-conjugated donkey-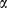-goat IgG (Dianova, Hamburg, Germany) was followed by chemiluminescent detection (Pierce). Signals on blots were quantified with ImageJ.

## Supporting Information

Text S1 **Additional computational and experimental results.** Figures and tables giving detailed computational and experimental results.
(PDF)
