# Supplementary material for: Signaling Domain of Sonic Hedgehog as Cannibalistic Calcium-Regulated Zinc-Peptidase
Source: PLoS Comput Biol. 2014 Jul 17;10(7):e1003707. doi: 10.1371/journal.pcbi.1003707 (PMC4102407; doi:10.1371/journal.pcbi.1003707)
Supplement: Text S1 — Additional computational and experimental results. Figures and tables giving detailed computational and experimental results. (PDF) [file pcbi.1003707.s001.pdf]

# Supporting Information for:

# Signaling Domain of Sonic Hedgehog as Cannibalistic Calcium-Regulated Zinc-Peptidase

Rocio Rebollido-Rios[1], Shyam Bandari[3], Christoph Wilms[1], Stanislav Jakuschev[1], Andrea Vortkamp[2], Kay Grobe[3], Daniel Hoffmann[1,*]
**1 Research Group Bioinformatics, Faculty of Biology, Center of Medical Biotechnology, University of Duisburg-Essen, Essen, Germany**
**2 Department of Developmental Biology, Faculty of Biology, Center of Medical Biotechnology, University of Duisburg-Essen, Essen, Germany**
**3 Institute of Physiological Chemistry and Pathobiochemistry, Faculty of Medicine, University of Münster, Münster, Germany**
**∗ E-mail: kgrobe@uni-muenster.de, daniel.hoffmann@uni-due.de**

## List of Figures


1 B-factors . . . . . . . . . . . . . . . . . . . . . . . . . . . . 2
2 Backbone RMSDs . . . . . . . . . . . . . . . . . . . . . . . . 3
3 Calcium binding and zinc center geometry . . . . . . . . . . 4
4 Docking of cholesterol . . . . . . . . . . . . . . . . . . . . . . 5
5 Distance of $Zn^{2+}$ and water . . . . . . . . . . . . . . . . . . . . 6
6 Water, zinc, ligand angle . . . . . . . . . . . . . . . . . . . . . 7
7 Electrostatics at CDO binding site . . . . . . . . . . . . . . . . 8
8 Zinc and flexibility . . . . . . . . . . . . . . . . . . . . . . . . 9
9 ShhN mutant stabilities at pH 5 and 6 . . . . . . . . . . . . . . 10


## List of Tables


1 RMSF and calcium state . . . . . . . . . . . . . . . . . . . . . 11
2 RMSDs between LAS enzymes and ShhN . . . . . . . . . . . 11
3 Comparison with 2vo9 . . . . . . . . . . . . . . . . . . . . . . 11
4 Comparison with 1u10 . . . . . . . . . . . . . . . . . . . . . . 12
5 Comparison with 1r44 . . . . . . . . . . . . . . . . . . . . . . 12
6 Comparison between MDs of LAS 1lbu and ShhN . . . . . . 13
7 MDs of LAS 2vo9 vs. ShhN . . . . . . . . . . . . . . . . . . . 13
8 Distances of E127 and H135 . . . . . . . . . . . . . . . . . . . 14

9 Distances of H135 and E177 . . . . . . . . . . . . . . . . . . . . 14
10 Docking without catalytic water . . . . . . . . . . . . . . . . . 15
11 Docking with catalytic water . . . . . . . . . . . . . . . . . . . 16
12 pKa of calcium ligands . . . . . . . . . . . . . . . . . . . . . . . 17
13 Position of Zn-co-ordinating water . . . . . . . . . . . . . . . 17
14 in vitro results . . . . . . . . . . . . . . . . . . . . . . . . . . . 18
15 Primer sequences . . . . . . . . . . . . . . . . . . . . . . . . . . 19

# Figures

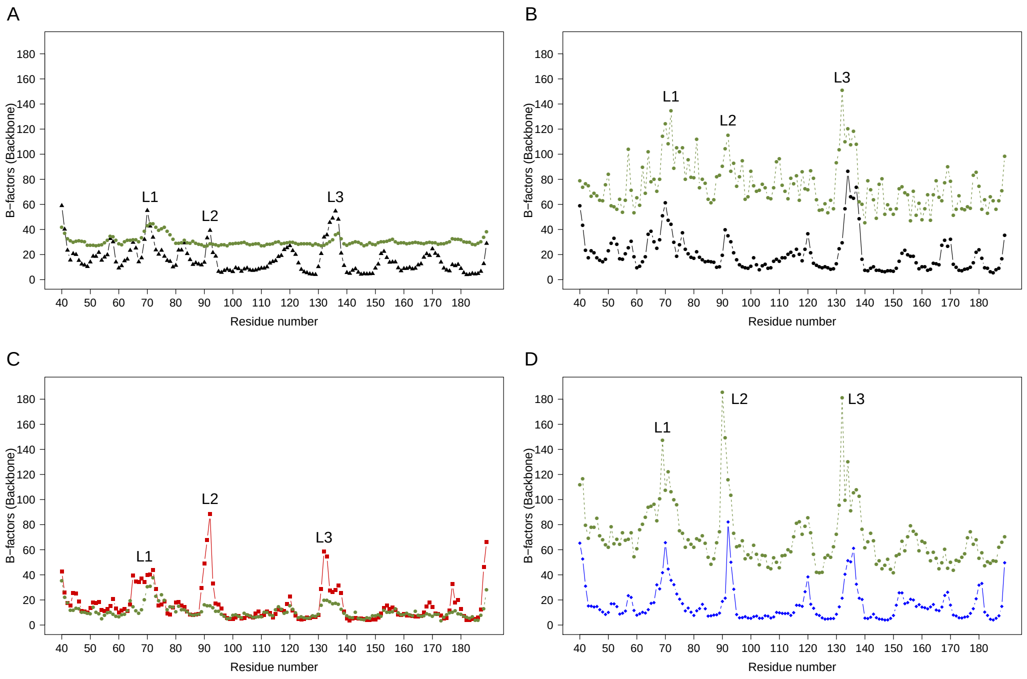


Figure 1: B-factors from molecular dynamics simulations of ShhN in states $Ca2_{Ihog}$ (A), $Ca2_{Hhip}$ (B), $Ca0$ (C), and $Ca1$ (D). The green curves show the corresponding experimental B-factors.

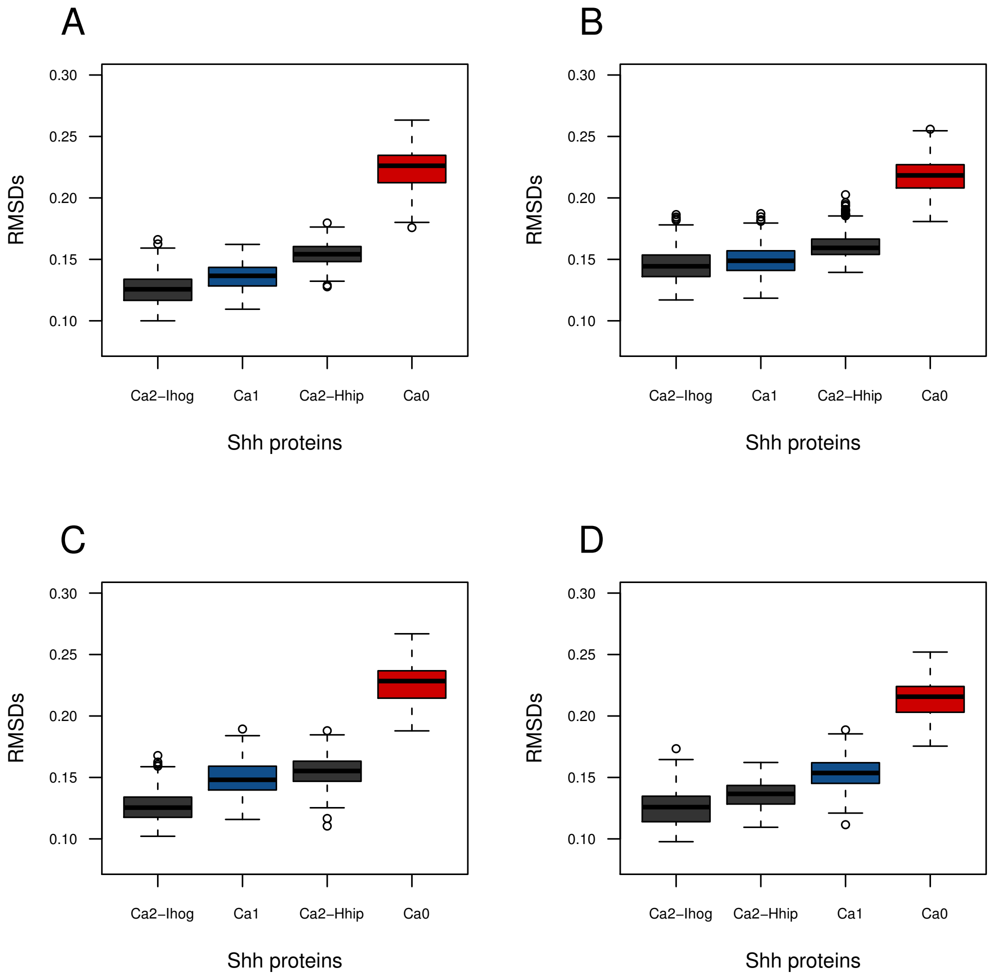


Figure 2: Backbone RMSDs between, on one hand, the four different X-ray structures representative of $Ca2_{Ihog}$ (3d1m, panel A), $Ca2_{Hhip}$ (2wfx, B), $Ca0$ (1vhh, C), $Ca1$ (3n1r, D), and, on the other hand, MD trajectories of ShhN in these states; e.g. the first boxplot (grey) in panel A gives the RMSD between 3d1m and an MD trajectory based on 3d1m. Boxplots within each of the four panels are sorted according to ascending median of RMSD.

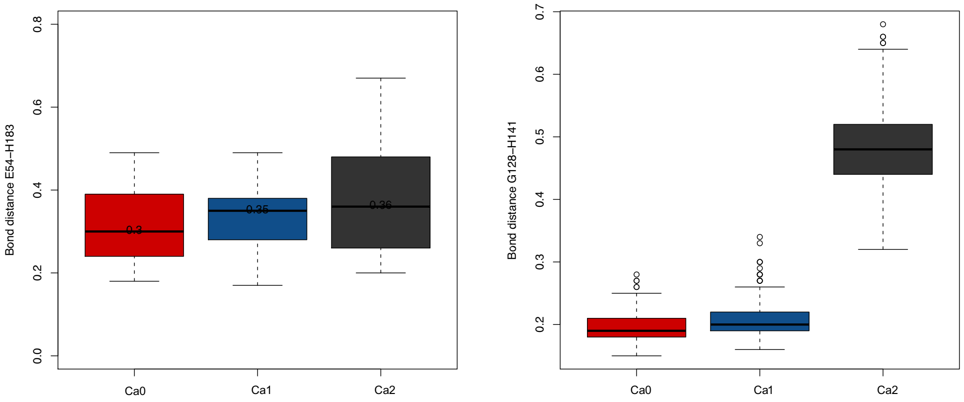


Figure 3: Effect of calcium binding on geometry of interactions that stabilize the zinc center. Shown are the distributions of distances (in nm; sampled by MD simulations) between carboxylate or carbonyl groups and histidine imidazole rings that co-ordinate the zinc. Left: the distance between groups from E54 and H183, both distal to the $Ca^{2+}$ binding site, is barely affected by $Ca^{2+}$ binding. Right: Binding of the $Ca2$ calcium ion breaks the hydrogen bond between G128 carbonyl and H141 imidazole N-H.

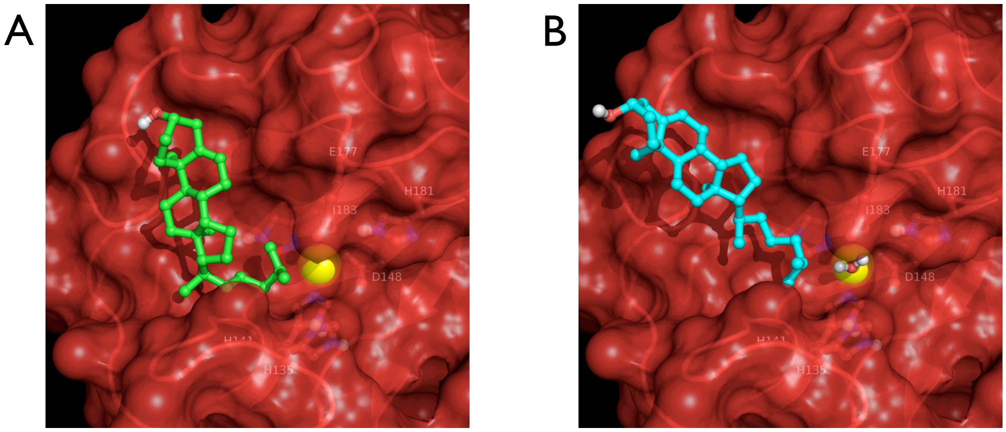


Figure 4: Docking of cholesterol to ShhN (1vhh), without zinc co-ordinating water (A), and with zinc co-ordinating water (B). The best (green) and second best (blue) poses are shown.

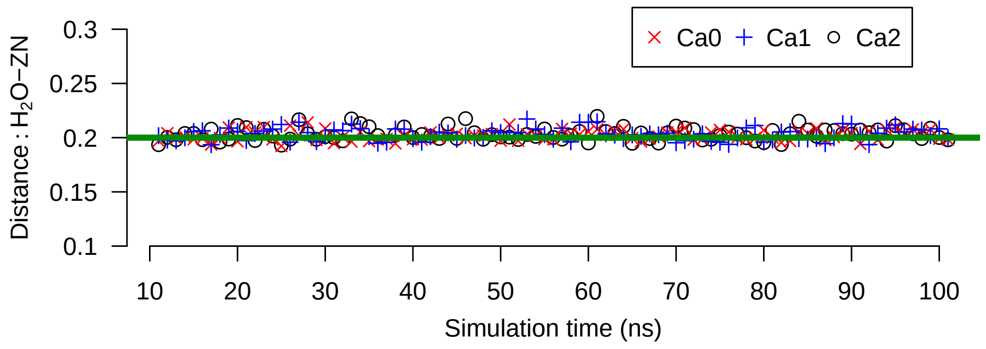


Figure 5: Distance between zinc ion and oxygen of water molecule close to the position of the putative catalytic water.

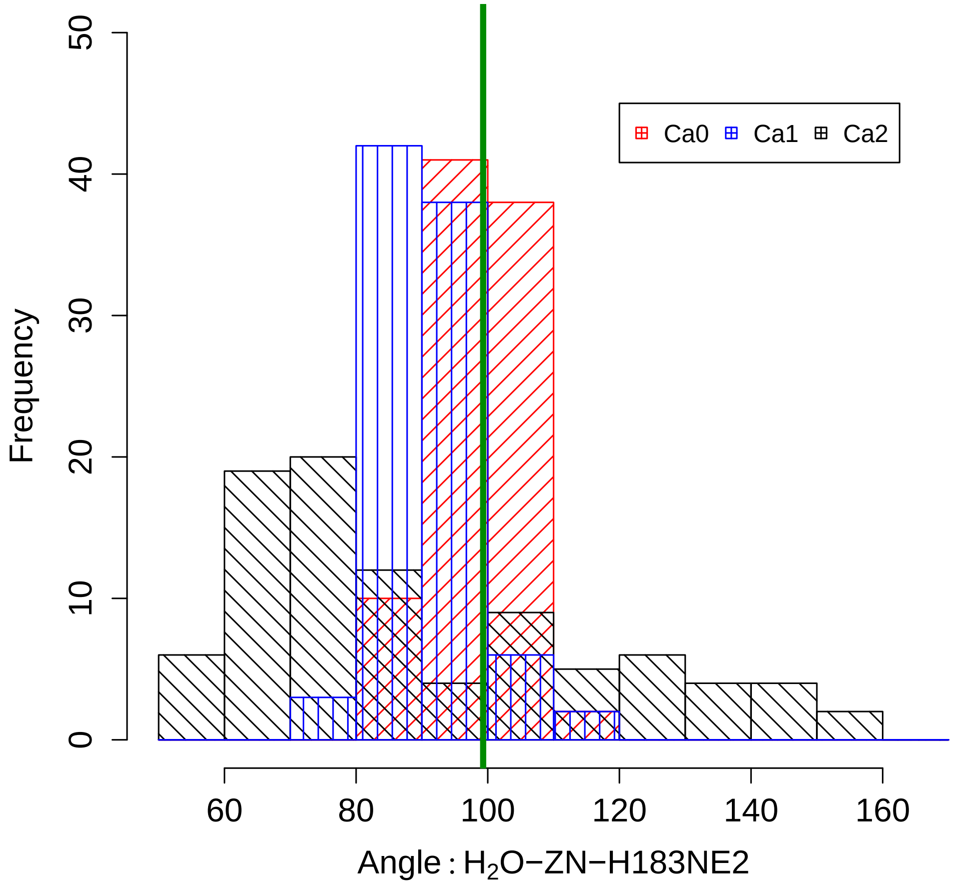


Figure 6: Histogram of angle (in degrees) between oxygen of putative catalytic water, zinc ion, and zinc ligand $N_{\delta 1}$ of H183 as sampled by MD simulations. The green line marks the angle measured in the X-ray structure 1vhh [1].

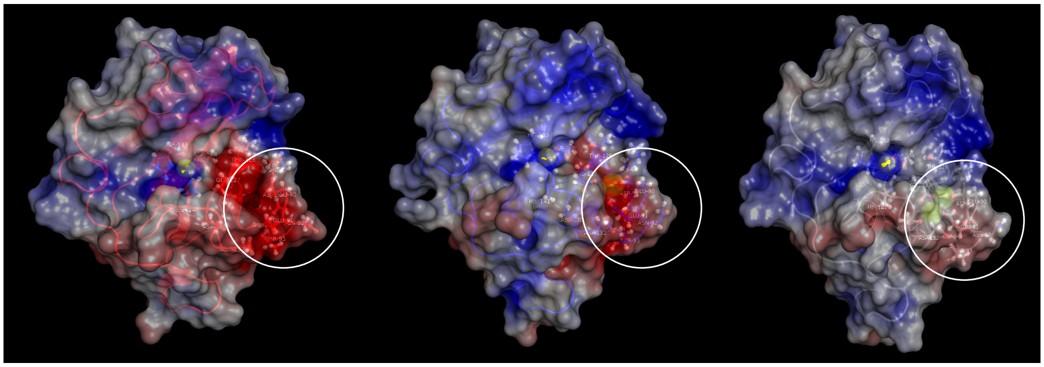

Figure 7: Electrostatics of ShhN states Ca0 (left), Ca1 (center), and Ca2 (right). Electrostatic potential on the surface of ShhN is color-coded between high (blue) and low (red). The circle encloses the loop where the calcium ions are bound, which is approximately the binding site of CDO. Calcium and zinc ions are depicted as green and yellow spheres, respectively. Electrostatic potential were scaled to the range of -5 (red) and 5 kT/e (blue).

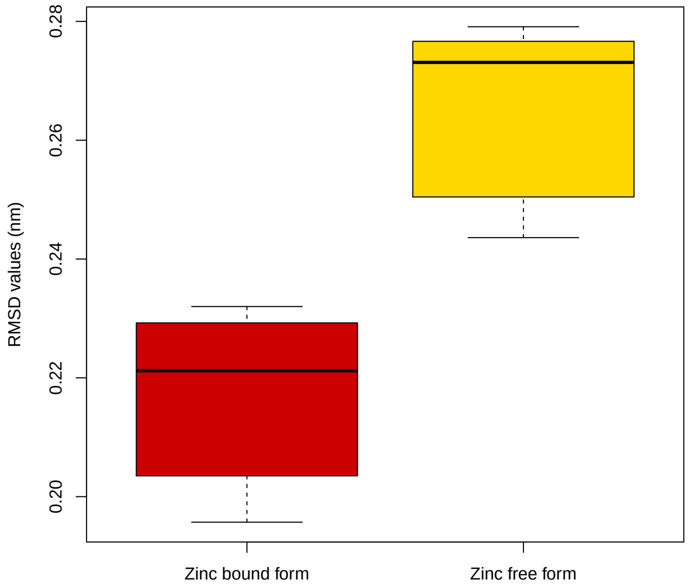


Figure 8: Effect of zinc ion on RMSD based on PDB entry 1vhh. The RMSD (in nm) of the structure with $Zn^{2+}$ (left boxplot) is clearly lower than that without $Zn^{2+}$ (right boxplot), in agreement with experiment [2]. Whiskers and edges of boxplots mark quartiles of sampled RMSD values, bold bar in the colored boxes is the median.

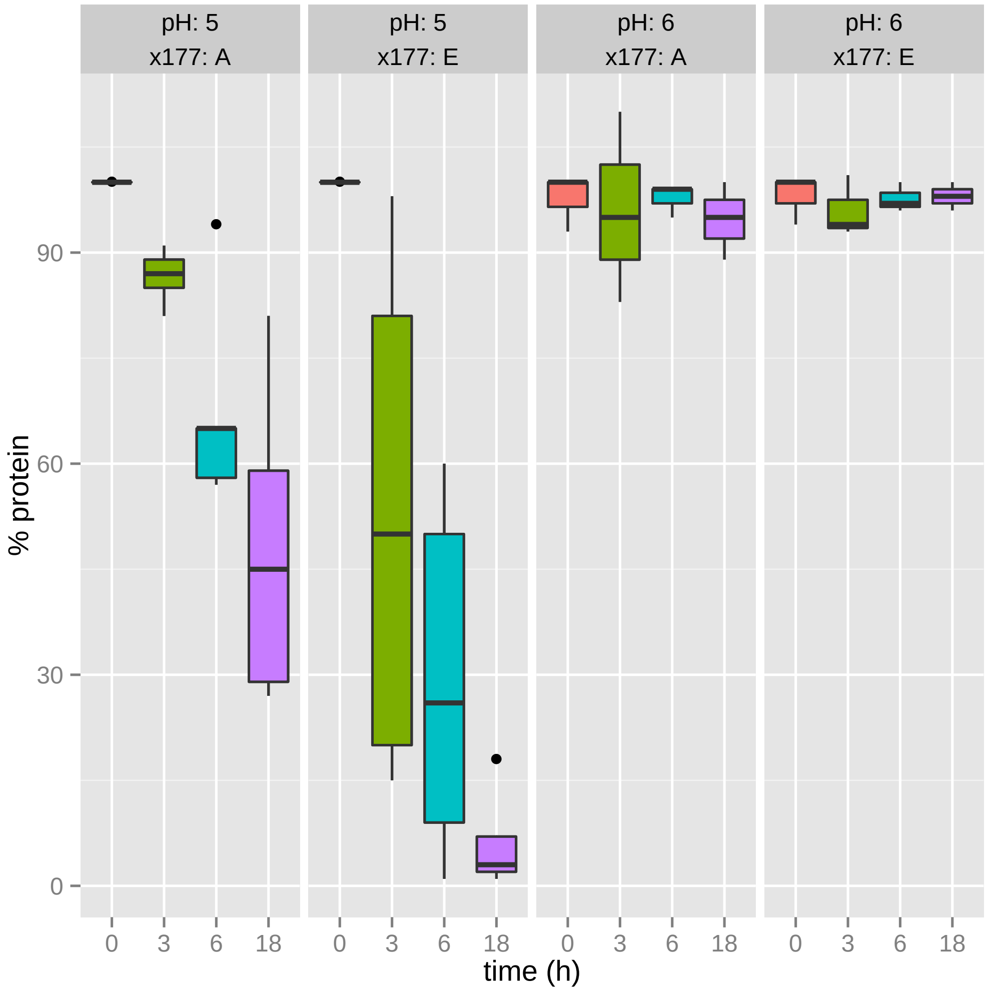


Figure 9: *In vitro* tests of ShhN mutant stabilities against proteolysis at pH 5 and pH 6. The content of ShhN mutant proteins $Pep^{+}$ (E177) and $Pep^{-}$ (E177A) over time is shown relative to the maximum protein content of the respective measurement series (data as in Table 15 of this supporting information). pH is 5 in the first two panels and 6 in the last two. The first and third panel refer to the E177A mutant $Pep^{-}$, the second and fourth to the mutant $Pep^{+}$ with wild type catalytic E177. Box elements (upper and lower whisker, upper and lower box) at each time point indicate quartiles of measured values, single points are outliers. Colors encode time points for easier comparison between panels.

# Tables

| | Mean RMSF (nm) | p-values | | |
|---|---|---|---|---|
| | | $Ca1$ | $Ca2_{Hhip}$ | $Ca2_{Ihog}$ |
| $Ca0$ | 0.12 | $8.8 \cdot 10^{-15}$ | $5.4 \cdot 10^{-11}$ | $4.3 \cdot 10^{-12}$ |
| $Ca1$ | 0.08 | - | 0.04 | 0.07 |
| $Ca2_{Hhip}$ | 0.09 | - | - | 0.87 |
| $Ca2_{Ihog}$ | 0.09 | - | - | - |

Table 1: Mean values of RMSFs from Figure 1 of main text, and p-values from Wilcoxon rank sum tests with null hypotheses that there is no shift between RMSF distributions in the compared pairs of states.

| | p-values | | |
|---|---|---|---|
| | $Ca1$ | $Ca2_{Hhip}$ | $Ca2_{Ihog}$ |
| $Ca0$ | 0.13 | $2.5 \cdot 10^{-15}$ | $4.9 \cdot 10^{-8}$ |
| $Ca1$ | - | $2.2 \cdot 10^{-16}$ | $1.9 \cdot 10^{-12}$ |
| $Ca2_{Hhip}$ | - | - | 0.04 |

Table 2: P-values of RMSD comparisons (Wilcoxon tests) between zinc center of LAS enzyme X-ray structure 1lbu and zinc centers of ShhN in MD simulations of states $Ca0$, $Ca1$, $Ca2_{Hhip}$, and $Ca2_{Ihog}$ (see Figure 4A of main text).

| | p-values | | |
|---|---|---|---|
| | $Ca1$ | $Ca2_{Hhip}$ | $Ca2_{Ihog}$ |
| $Ca0$ | 0.05 | $2.2 \cdot 10^{-16}$ | $5.5 \cdot 10^{-13}$ |
| $Ca1$ | - | $2.2 \cdot 10^{-16}$ | $1.5 \cdot 10^{-14}$ |
| $Ca2_{Hhip}$ | - | - | 0.9 |

Table 3: As Table 2, but referring to X-ray structure of LAS enzyme 2vo9.

| | p-values | | |
|---|---|---|---|
| | $Ca1$ | $Ca2_{Hhip}$ | $Ca2_{Ihog}$ |
| $Ca0$ | 0.5 | $8.1 \cdot 10^{-11}$ | $1.1 \cdot 10^{-10}$ |
| $Ca1$ | - | $1.6 \cdot 10^{-15}$ | $3.0 \cdot 10^{-15}$ |
| $Ca2_{Hhip}$ | - | - | 0.8 |

Table 4: As Table 2, but referring to X-ray structure of LAS enzyme 1u10.

| | p-values | | |
|---|---|---|---|
| | $Ca1$ | $Ca2_{Hhip}$ | $Ca2_{Ihog}$ |
| $Ca0$ | 0.3 | $2.2 \cdot 10^{-16}$ | $1.6 \cdot 10^{-15}$ |
| $Ca1$ | - | $2.2 \cdot 10^{-16}$ | $1.3 \cdot 10^{-13}$ |
| $Ca2_{Hhip}$ | - | - | 0.9 |

Table 5: As Table 2, but referring to X-ray structure of LAS enzyme 1r44.

| | p-values | | |
|---|---|---|---|
| | $Ca1$ | $Ca2_{Hhip}$ | $Ca2_{Ihog}$ |
| $Ca0$ | 0.2 | $3.3 \cdot 10^{-11}$ | $2.1 \cdot 10^{-10}$ |
| $Ca1$ | - | $2.2 \cdot 10^{-16}$ | $4.9 \cdot 10^{-16}$ |
| $Ca2_{Hhip}$ | - | - | 0.14 |

Table 6: P-values of RMSD comparisons (Wilcoxon tests) between zinc center of LAS enzyme structure 1lbu and zinc centers of ShhN in states $Ca0$, $Ca1$, $Ca2_{Hhip}$, and $Ca2_{Ihog}$ (see Figure 4B of main text). In contrast to Table 2, the structures of the zinc centers of both the LAS enzyme and ShhN are taken from MD simulations of the respective proteins.

| | p-values | | |
|---|---|---|---|
| | $Ca1$ | $Ca2_{Hhip}$ | $Ca2_{Ihog}$ |
| $Ca0$ | 0.2 | $2.5 \cdot 10^{-12}$ | $2.2 \cdot 10^{-16}$ |
| $Ca1$ | - | $1.4 \cdot 10^{-12}$ | $2.2 \cdot 10^{-16}$ |
| $Ca2_{Hhip}$ | - | - | 0.4 |

Table 7: As Table 6, but for comparison of LAS enzyme 2vo9 and ShhN.

| $Ca^{2+}$ state | Min. | 1st Qu. | Median | Mean | 3rd Qu. | Max. |
|---|---|---|---|---|---|---|
| $Ca0$ | 0.129 | 0.181 | 0.194 | 0.199 | 0.216 | 0.270 |
| $Ca2$ | 0.157 | 0.179 | 0.192 | 0.195 | 0.207 | 0.290 |

Table 8: Distribution of distances (in nm) between carboxylate-O of E127 and imidazole proton of H135, along the charged hydrogen bond between these groups. Given are the mean distance and the distances demarcating the quartiles, both for $Ca0$ and $Ca2$, corresponding to vertical axis of Figure 5B of main text. There is little change between the states, i.e. the H-bond is conserved.

| $Ca^{2+}$ state | Min. | 1st Qu. | Median | Mean | 3rd Qu. | Max. |
|---|---|---|---|---|---|---|
| $Ca0$ | 0.675 | 0.733 | 0.758 | 0.768 | 0.788 | 1.000 |
| $Ca2$ | 0.653 | 0.788 | 0.827 | 0.848 | 0.878 | 1.150 |

Table 9: Distribution of distances (in nm) between side chains of H135 and E177, the "catalytic clamp". Given are the mean distance and the distances demarcating the quartiles, both for $Ca0$ and $Ca2$, corresponding to horizontal axis of Figure 5B of main text. The comparison shows that the clamp opens from $Ca0$ to $Ca2$, and that it becomes more flexible.

| Modes | Affinity (kcal/mol) | RMSD l.b | RMSD u.b |
|---|---|---|---|
| 1 | -6.8 | 0.000 | 0.000 |
| 2 | -6.7 | 2.452 | 3.960 |
| 3 | -6.5 | 1.887 | 3.633 |
| 4 | -6.2 | 3.433 | 5.034 |
| 5 | -6.1 | 2.102 | 3.352 |
| 6 | -6.0 | 3.974 | 6.015 |
| 7 | -6.0 | 2.316 | 3.153 |
| 8 | -5.9 | 3.329 | 7.969 |
| 9 | -5.9 | 2.378 | 3.750 |
| 10 | -5.9 | 2.786 | 5.661 |
| 11 | -5.8 | 2.734 | 7.353 |
| 12 | -5.8 | 2.944 | 7.585 |
| 13 | -5.6 | 2.186 | 3.240 |
| 14 | -5.6 | 20.878 | 24.203 |
| 15 | -5.5 | 20.929 | 24.506 |
| 16 | -5.5 | 5.170 | 7.745 |
| 17 | -5.5 | 4.188 | 6.568 |
| 18 | -5.5 | 4.828 | 7.686 |
| 19 | -5.4 | 3.379 | 5.252 |
| 20 | -5.3 | 1.940 | 3.100 |

Table 10: Summary of docking results of cholesterol to the surface of ShhN, without zinc co-ordinating water molecule. Affinity (kcal/mol): the affinity estimate by AutoDock vina. The two RMSD columns give the root mean square deviation between the respective docking pose and the docking pose with best affinity (top ranking pose); “l.b” is a lower bound of RMSD, considering matches between atoms of same type, while “u.b” is an upper bound, computed for matches between exactly equivalent atoms according to the chosen numbering of atoms in the ligand.

| Modes | Affinity (kcal/mol) | RMSD l.b | RMSD u.b |
|---|---|---|---|
| 1 | -7.2 | 0.000 | 0.000 |
| 2 | -7.1 | 2.400 | 3.725 |
| 3 | -7.0 | 1.461 | 2.358 |
| 4 | -6.8 | 5.002 | 6.808 |
| 5 | -6.7 | 1.545 | 3.144 |
| 6 | -6.5 | 2.334 | 4.185 |
| 7 | -6.4 | 1.579 | 3.111 |
| 8 | -6.4 | 2.344 | 3.223 |
| 9 | -6.3 | 1.941 | 2.343 |
| 10 | -6.2 | 2.393 | 4.236 |
| 11 | -6.2 | 1.668 | 2.152 |
| 12 | -6.1 | 2.319 | 3.742 |
| 13 | -6.1 | 3.150 | 5.073 |
| 14 | -6.0 | 21.800 | 26.178 |
| 15 | -5.9 | 3.228 | 4.485 |
| 16 | -5.9 | 21.796 | 25.745 |
| 17 | -5.9 | 22.408 | 26.172 |
| 18 | -5.8 | 22.385 | 24.987 |
| 19 | -5.7 | 3.241 | 8.804 |
| 20 | -5.7 | 1.651 | 2.500 |

Table 11: Same as Table 10, but including the zinc co-ordinating water molecule as in X-ray structure 1vhh.

| Residues | Ca0 state | Ca2 state |
|---|---|---|
| E90 | 3.55 | 2.16 |
| E91 | 5.40 | 5.96 |
| D96 | 6.00 | 5.44 |
| E127 | 5.02 | 5.97 |
| D130 | 3.46 | 2.17 |
| D132 | 4.37 | 2.37 |

Table 12: Predicted $pK_a$ values for calcium ligands in Ca0 (1vhh) and Ca2 states (3d1m).

| PDB entries | Ligands | | | Angles | | |
|---|---|---|---|---|---|---|
| | X | Y | Z | O-Zn-X | O-Zn-Y | O-Zn-Z |
| 1vhh | H141 | D148 | H183 | 111.1° | 116.3° | 99.3° |
| 1lbu | H154 | D161 | H197 | 121.9° | 115.4° | 97.6° |
| 2vo9 | H80 | D87 | H133 | 121.6° | 107.4° | 102.9° |
| 1r44 | H116 | D123 | H184 | 106.5° | 126.9° | 97.9° |
| 8tln | H142 | H146 | E166 | 114.2° | 120.3° | 99.3° |

Table 13: The position of the Zn-co-ordinating water in ShhN (1vhh), three LAS enzymes (1lbu, 2vo9, 1r44), and thermolysin (8tln). Angles refer to positions of water oxygen, zinc ion, and closest oxygen (Asp, Glu) or nitrogen (His) atoms of co-ordinating amino acids.

Table 14: Results of *in vitro* experiments with mutants $Pep^+$ (x177=E) and $Pep^-$ (x177=A). Time is given in hours and protein is percent of maximum protein content in the respective experiment.

```
x177,pH,time,protein
E,6,0,100
E,6,0,94
E,6,0,100
E,5,0,100
E,5,0,100
E,5,0,100.1
E,5,0,100
E,5,0,100
A,6,0,100
A,6,0,100
A,6,0,93
A,5,0,100
A,5,0,100
A,5,0,100.1
A,5,0,100
A,5,0,100
E,6,3,101
E,6,3,94
E,6,3,93
E,5,3,50
E,5,3,98
E,5,3,81
E,5,3,20
E,5,3,15
A,6,3,95
A,6,3,110
A,6,3,83
A,5,3,91
A,5,3,85
A,5,3,89
A,5,3,81
A,5,3,87
```

```
x177,pH,time,protein
E,6,6,96
E,6,6,100
E,6,6,97
E,5,6,26
E,5,6,60
E,5,6,50
E,5,6,9
E,5,6,1
A,6,6,95
A,6,6,99
A,6,6,99
A,5,6,65
A,5,6,94
A,5,6,65
A,5,6,58
A,5,6,57
E,6,18,96
E,6,18,98
E,6,18,100
E,5,18,3
E,5,18,18
E,5,18,7
E,5,18,2
E,5,18,1
A,6,18,95
A,6,18,100
A,6,18,89
A,5,18,45
A,5,18,81
A,5,18,59
A,5,18,27
A,5,18,29
```

Table 15: Primer sequences for $Pep^{+}$ and $Pep^{-}$ mutants.

```
Catalytic residue knockout mutant primers:

E177A_Sense:
CGACTGGGTCTACTATGCATCCAAAGCTCACATCC

E177A_Antisense:
GGATGTGAGCTTTGGATGCATAGTAGACCCAGTCG


Calcium binding pocket mutant primers:

E90 and 91A_Sense:
CGACATCATATTTAAGGATGCGGCAAACACGGGAGCAGACC

E90 and 91A_Antisense:
GGTCTGCTCCCGTGTTTGCCGCATCCTTAAATATGATGTCG

E127A_Sense:
CTGCGAGTGACCGCGGGCTGGGATGAG

E127A_Antisense:
CTCATCCCAGCCCGCGGTCACTCGCAG
```

# References


1. Hall TM, Porter JA, Beachy PA, Leahy DJ (1995) A potential catalytic site revealed by the 1.7-Å crystal structure of the amino-terminal signalling domain of sonic hedgehog. Nature 378: 212-6.

2. Day ES, Wen D, Garber EA, Hong J, Avedissian LS, et al. (1999) Zinc-dependent structural stability of human sonic hedgehog. Biochemistry 38: 14868-80.
